# Intradiscal Mesenchymal Stromal Cell Therapy for the Treatment of Low Back Pain Due to Moderate‐to‐Advanced Multilevel Disc Degeneration: A Preliminary Report of a Double‐Blind, Phase IIB Randomized Clinical Trial (DREAM Study)

**DOI:** 10.1002/jsp2.70086

**Published:** 2025-06-02

**Authors:** Gianluca Vadalà, Fabrizio Russo, Cristiana Lavazza, Giorgia Petrucci, Luca Ambrosio, Silvia Budelli, Elisa Montelatici, Giuseppina Di Giacomo, Claudia Cicione, Veronica Tilotta, Giuseppe Francesco Papalia, Matteo Pileri, Amalia Bruno, Salvatore La Rosa, Emilio Carrino, Eliodoro Faiella, Massimiliano Carassiti, Lorenza Lazzari, Rocco Papalia, Vincenzo Denaro

**Affiliations:** ^1^ Operative Research Unit of Orthopaedic and Trauma Surgery Fondazione Policlinico Universitario Campus Bio‐Medico Rome Italy; ^2^ Laboratory for Regenerative Orthopaedics, Research Unit of Orthopaedic and Trauma Surgery Department of Medicine and Surgery, Università Campus Bio‐Medico di Roma Rome Italy; ^3^ Unit of Cell and Gene Therapies Fondazione IRCCS Ca' Granda Ospedale Maggiore Policlinico of Milano Milan Italy; ^4^ Operative Research Unit of Radiology and Interventional Radiology Fondazione Policlinico Universitario Campus Bio‐Medico Rome Italy; ^5^ Research Unit of Radiology Department of Medicine and Surgery, Università Campus Bio‐Medico di Roma Rome Italy; ^6^ Operative Research Unit of Anesthesia and Intensive Care Fondazione Policlinico Universitario Campus Bio‐Medico Rome Italy; ^7^ Research Unit of Anaesthesia and Intensive Care Department of Medicine and Surgery, Università Campus Bio‐Medico di Roma Rome Italy

**Keywords:** clinical trial, intervertebral disc degeneration, intervertebral disc regeneration, low back pain, RCT, regenerative medicine, stem cells

## Abstract

**Background:**

Low back pain (LBP) is a leading cause of disability worldwide, often associated with intervertebral disc degeneration (IDD). Mesenchymal stromal cells (MSCs) have emerged as a promising regenerative therapy for IDD due to their ability to promote tissue repair. This phase IIB randomized controlled trial aimed to evaluate the safety and efficacy of autologous bone marrow‐derived MSC (BM‐MSC) intradiscal injections in patients with chronic LBP due to moderate‐to‐advanced multilevel IDD.

**Methods:**

Fifty‐two patients with chronic LBP unresponsive to conservative treatments with moderate‐to‐advanced IDD at up to three lumbar levels were included. Participants were randomized to receive either BM‐MSCs or a sham procedure. Clinical outcomes, including pain intensity (VAS), disability (ODI), and quality of life (SF‐36), were assessed at baseline, 1‐, 3‐, and 6‐months postinjection. Structural changes were evaluated via MRI using Pfirrmann grading, disc height index (DHI), and T2 mapping at baseline, 3 and 6 months.

**Results:**

Of the 52 enrolled patients, 46 completed the 6‐month follow‐up (BM‐MSC group: *n* = 21; sham group: *n* = 25). BM‐MSC injections were well‐tolerated, with no major adverse events reported. Structural improvements were observed in the BM‐MSC group, including significant increases in DHI and nonsignificant improvements in T2 relaxation times at 3 and 6 months. Modified Pfirrmann grades showed transient improvement at 3 months but returned to baseline at 6 months. Despite these radiological changes, clinical outcomes such as VAS, ODI, and SF‐36 scores improved similarly in both groups without significant intergroup differences at any timepoint. The sham group demonstrated slightly greater improvements in disability (ODI) and physical quality‐of‐life scores (SF‐36 PCS).

**Conclusions:**

Autologous BM‐MSC intradiscal injection is a safe and promising approach in patients with chronic LBP due to moderate‐to‐advanced multilevel IDD. However, despite these regenerative effects, no significant clinical advantages over the sham procedure were observed within 6 months of follow‐up.

## Graphical abstract

This phase IIB randomized controlled trial evaluated the safety and efficacy of intradiscal injections of autologous bone marrow‐derived mesenchymal stromal cells (BM‐MSCs) in patients with chronic low back pain from moderate‐to‐advanced multilevel disc degeneration. While the BM‐MSC treatment was safe and produced significant improvements in disc structure (notably an increased disc height index), these radiological benefits did not translate into superior clinical outcomes—such as reduced pain or disability—compared to a sham procedure over a 6‐month follow‐up period.

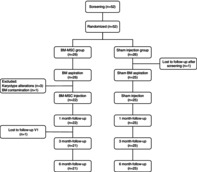

## 1 Introduction

Low back pain (LBP) is the leading cause of years lived with disability (YLDs) in the world, affecting up to 90% of adults at least once in their lifetime. While most cases are transient and resolve with conservative treatment, approximately 10% become chronic, significantly impairing functionality and quality of life, imposing a substantial socioeconomic burden [1].

Chronic LBP is frequently associated with intervertebral disc degeneration (IDD), a progressive, multifactorial condition characterized by structural deterioration of the intervertebral disc (IVD), ultimately leading to biomechanical dysfunction and complications such as disc herniation, spinal stenosis, and degenerative spondylolisthesis. Pathophysiologically, IDD is hallmarked by the gradual depletion of nucleus pulposus (NP) cells and a shift toward catabolic metabolism, characterized by increased matrix metalloproteinase (MMP) activity, reduced disc cell viability, and decreased proteoglycan synthesis [2].

Over the past few decades, several preclinical and preliminary clinical studies have explored the potential of mesenchymal stromal cells (MSCs) as a regenerative therapy for IDD [3]. MSCs are multipotent adult cells with clonogenic potential, self‐renewal capacity, and the ability to differentiate into various cell types, including those residing in the bone, cartilage, muscle, and adipose tissues [4]. These cells can be easily harvested from multiple sources, such as bone marrow (BM), umbilical cord, cord blood, and subcutaneous fat, and possess low immunogenicity and resilience in harsh microenvironments, making them well‐suited for cell‐based therapies [5, 6, 7, 8].

Within the degenerative disc microenvironment, MSCs have been shown to differentiate into NP‐like cells and enhance native cell anabolism by secreting growth factors and anti‐inflammatory cytokines, ultimately promoting tissue repair [5, 9, 10]. This ability to restore disc cellularity and shift metabolism toward regeneration has driven growing interest in intradiscal MSC therapy [3]. Recent clinical trials, including the RESPINE study [11] and the randomized controlled trial (RCT) by Amirdelfan et al. [12], have demonstrated the safety and efficacy of allogeneic bone marrow‐derived MSCs (BM‐MSCs) for single‐level IDD, showing a low incidence of adverse events (AEs), improvements in pain and disability scores, with no clinically relevant difference compared to controls. Moreover, despite the advantages of allogeneic MSCs as an off‐the‐shelf product, concerns persist regarding potential immune reactions following transplantation into the host [13]. Additionally, these studies mainly included patients with single‐level, mild‐to‐moderate IDD, with limited evidence regarding the application of this therapy in multi‐level and more advanced degeneration.

This phase IIB randomized controlled trial was conducted to evaluate the safety and efficacy of a single intradiscal injection of autologous BM‐MSCs for single‐ or multilevel moderate‐to‐advanced IDD. Here, we present preliminary clinical and radiological outcomes at an intermediate follow‐up of 6 months.

## 2 Materials and Methods

### 2.1 Study Design

This was a single‐center, prospective, randomized, controlled, double‐blinded phase IIb trial in which patients were assigned to receive either an intradiscal injection of autologous BM‐MSCs or a sham intramuscular injection. The study was conducted according to the principles stated by the Declaration of Helsinki, approved by the local institutional review board (IRB) under the agreement n. ComEt CBM 32.19, and registered in the ClinicalTrials.gov (ID: NCT05066334) and EudraCT (ID: 2019‐002749‐40) databases. The study was written according to the Consolidated Standards of Reporting Trials (CONSORT) reporting guideline [14]. All participants provided written informed consent. The full study protocol is available as Supporting Information.

### 2.2 Study Participants

Participants were recruited by orthopaedic surgeons at Fondazione Policlinico Universitario Campus Bio‐Medico (Rome, Italy). Eligible patients were aged between 18 and 65 years and had chronic LBP (≥ 6 months, baseline average visual analogue scale [VAS] = 40 mm) unresponsive to conventional conservative treatments (e.g., analgesics, physiotherapy, bracing) and associated with moderate IDD (modified Pfirrmann grades 3–7 [15]) at a maximum of three lumbar IVDs. The diagnosis of chronic discogenic LBP was made based on a combination of patient history, clinical examination, and imaging assessment. No provocative discography was performed to select the symptomatic level(s). Exclusion criteria included spinal deformity, infection, symptomatic facet joint syndrome, spinal stenosis, instability, lumbar disc herniation with sciatica, endplate abnormalities, and Modic changes type 2 or 3.

### 2.3 Randomization and Masking

After baseline assessments, patients were randomly assigned to receive intradiscal injection(s) of autologous BM‐MSCs in the affected disc(s) or a sham procedure in a 1:1 ratio. Randomization was conducted by an independent contract research organization (CRO) using a centralized computer‐generated system with varying block sizes (two to four). Treatment allocation was planned to remain concealed until the last enrolled patient completes the 1‐year follow‐up. Only the pharmacists in charge of preparing the experimental therapeutic product and surgeons responsible for bone marrow (BM) aspiration and injections were aware of group assignments, while outcome assessors, radiological reviewers, investigators, and the biostatistician remained blinded. All the visits, procedures, and exams were performed at Fondazione Policlinico Universitario Campus Bio‐Medico.

### 2.4 Bone Marrow Aspiration

Patients were positioned prone under conscious sedation. After sterile draping, the posterior superior iliac spine (PSIS) was identified by manual palpation, and local anesthesia with 1% lidocaine was administered down to the periosteum. For patients randomized to undergo intradiscal cell injection, BM aspiration was performed using a 15G Jamshidi needle and biopsy trocar. Approximately 20 mL of BM were aspirated and aliquoted into five syringes, each containing 1 mL of 1000 U/mL heparin. In the sham group, the Jamshidi needle was advanced until contacting the PSIS surface, without penetrating the cortical bone. Eventually, a sterile dressing was applied. The harvested BM was transported to the Fondazione IRCCS Ca' Granda Ospedale Maggiore Policlinico (Milan, Italy) by a certified carrier under controlled conditions (validated cooler, 4°C–8°C, transport duration ≤ 24 h).

### 2.5 Cell Expansion

BM samples were accepted after verifying package integrity, sample‐patient ID matching, a collection volume of ≥ 15 mL, and negative results for mandatory biological tests (HBsAg, Anti‐HCV‐Ab, Anti‐HBc, Anti‐HIV‐1/2, and *Treponema pallidum* ). BM‐MSCs were isolated and expanded in a good manufacturing practice (GMP)‐classified facility approved by regulatory authorities, following a validated GMP‐compliant manufacturing protocol [16, 17]. Briefly, BM was seeded at 50 × 10^3^ cells/cm^2^ in a CellSTACK culture system (Macopharma) using alphaMEM complete medium (Macopharma) supplemented with 10% fetal bovine serum (Gibco, Thermo Fisher). The medium was refreshed every 3–4 days, and subculturing was performed on day 14 ± 1 by trypsinization (TrypLE Select, Gibco, Thermo Fisher Scientific). The final investigational medicinal product (IMP) was harvested on day 21 ± 1, packaged in 5‐mL Luer‐Lock syringes (Becton, Dickinson and Company) containing 2 mL of normal saline (Baxter) supplemented with 5% human serum albumin (Albumeon, CSL Behring). Quality control assessments were performed at three key production stages (starting material, intermediate product, and final product at days 0, 14 ± 1, and 21 ± 1), using validated analytical methods previously employed for other IMPs [18, 19]. Cell count (target dose), viability (≥ 80% propidium iodide‐negative cells), karyotyping (46,XX/46,XY), and colony‐forming unit fibroblast (CFU‐F) assays were assessed at each production stage. Sterility was assessed in the starting material and final product, whereas phenotyping (CD90, CD105, and CD73 expression; CD45 negativity) and bacterial endotoxin testing were conducted in the intermediate and final products. Eventually, Mycoplasma testing was performed on the final product only.

### 2.6 Intradiscal Injection

On the day of the injection, isolated BM‐MSCs were transported to the trial site through a certified carrier under controlled conditions (validated cooler, 4°C–8°C), appropriately stored by the local pharmacy, and injected within 24 h from shipment. The investigational product was provided in one or multiple 5‐mL Luer Lock syringes (one per disc level), each containing 15 ± 1 × 10^6^ BM‐MSCs cryopreserved in 2 mL of vehicle. The same number of syringes with the cell suspension or normal saline was put in an opaque cover and sleeve to maintain allocation concealment.

Patients were placed in the prone position on a radiolucent surgical table with a Wilson frame under deep sedation. After sterile draping, the optimal entry points for accessing the affected disc(s) were carefully identified under fluoroscopic guidance in anterior–posterior, lateral, and oblique views. Local anesthesia (1% lidocaine) was administered into the skin, subcutaneous tissue, and paravertebral muscles. Subsequently, a 23G Chiba needle was advanced along the planned trajectory under fluoroscopic guidance until penetrating the target NP. Additional multiplanar checks were performed to confirm accurate needle positioning. After removing the needle mandrel, the syringe containing the cell suspension was connected, and the product was slowly injected. The same procedure was repeated for each affected disc level. In the sham group, the procedure was identical, except that the Chiba needle was not advanced beyond the paravertebral muscles, and no injection was performed. A sterile dressing was applied at the end of the procedure.

### 2.7 Follow‐Up Assessments and Study Outcomes

Following the screening visit, patients were evaluated at 1, 3, and 6 months postinjection. At each follow‐up, clinical assessments were performed, including patient‐reported outcome measure (PROM) collection, blood sampling for routine laboratory tests, and urinalysis. Baseline imaging included standing lumbar x‐rays (anterior–posterior, lateral, and flexion‐extension views), while lumbar MRI scans were obtained at baseline, 3 months, and 6 months. Safety assessments involved monitoring for AEs, treatment‐emergent adverse events (TEAEs), and serious adverse events (SAEs), including infection, bleeding, and nerve injury with potential permanent damage. Additional safety evaluations included physical and neurological examinations, measurement of vital signs, review of concomitant medications, and imaging analysis. Patients were actively questioned about any new or worsening symptoms, signs, or conditions.

The primary outcomes of the study were improvements in LBP severity and disability. Pain intensity was measured using the VAS, ranging from 0 (no pain) to 100 (worst imaginable pain). Disability was assessed with the Oswestry Disability Index (ODI), a 10‐item questionnaire evaluating functional impairment across daily activities (e.g., pain intensity, personal care, lifting, walking, sitting, standing, sleeping, sex life, social life, and traveling), with scores ranging from 0% to 100%, where higher values indicate greater disability.

Secondary outcomes included improvements in quality of life, work ability, and structural changes in the IVD. Quality of life was assessed using the 36‐Item Short Form Survey (SF‐36), which evaluates eight health domains: physical functioning, role limitations due to physical and emotional health, energy/fatigue, emotional well‐being, social functioning, pain, general health perception, and health change, with higher scores indicating better health status. Work ability was evaluated with the Work Ability Index (WAI), which assesses work capacity related to one's lifetime best and work's demands, sick leaves, and work impairment due to disease. Structural disc changes were evaluated using lumbar 1.5 T MRI with T2‐weighted, T1 spin‐echo, short tau inversion recovery (STIR), and T2 mapping sequences. IDD severity was graded semiquantitatively using the modified Pfirrmann classification [15]. Disc height was measured using the disc height index (DHI), calculated as: 2 × (DH1 + DH2 + DH3)/(*A*1 + *A*2 + *A*3 + *B*1 + *B*2 + *B*3), where *A* and *B* represent the vertebral body lengths immediately above and below the target disc, respectively, and DH represents the disc height between adjacent vertebrae. Changes in disc height were expressed as a percentage of baseline values. IVD fluid content was quantitatively assessed by T2 mapping of target discs. Briefly, T2 maps and corresponding relaxation times were calculated for each region of interest (ROI) corresponding to the NP region of affected discs (Olea Sphere, v. 3.0, Olea Medical). Data were expressed as percent changes of T2 relaxation time values at each timepoint compared to baseline. All MRI data were blindly assessed by two independent reviewers with more than 5 years of experience in musculoskeletal radiology.

### 2.8 Sample Size Calculation

The sample size was determined to achieve 80% power with a two‐sided alpha of 5% in two balanced groups (1:1 ratio). Based on previous data [11], we expected a responder rate of 30% at 12 months in the control group and 68% in the MSC group, corresponding to an absolute difference of 38%. Using standard sample size calculations for a two‐group comparison of proportions, 23 participants per group are required. To account for an estimated 10% inclusion failure rate, the final sample size was set at 26 participants per group, for a total of 52 subjects.

### 2.9 Statistical Analysis

Continuous variables were represented as mean ± standard deviation (SD), whereas categorical variables were shown as counts and relative percentages. The normal distribution of analyzed data was assessed using the Wilk–Shapiro test. Between‐group comparisons were performed using the unpaired *t* test or the Mann–Whitney *U* test in the case of continuous variables and the chi‐square test or Fisher's exact test for categorical variables. Longitudinal evaluation of VAS, ODI, WAI, and SF‐36 scores according to treatment was assessed using mixed models for repeated measurements. Results were expressed as estimated mean or estimated mean difference from T0 with their 95% confidence interval (95% CI). Paired *t* test derived from linear mixed models was applied for within‐group comparisons. Two‐way ANOVA was utilized to assess the changes in mean modified Pfirrmann grades, DHI, and T2 relaxation times of target IVDs over time. *p*‐values < 0.05 were considered statistically significant. Statistical analysis was performed using Prism 10 (v. 10.4.1, GraphPad Software) and SAS software (v. 9.4, SAS Institute).

## 3 Results

**FIGURE 1:**
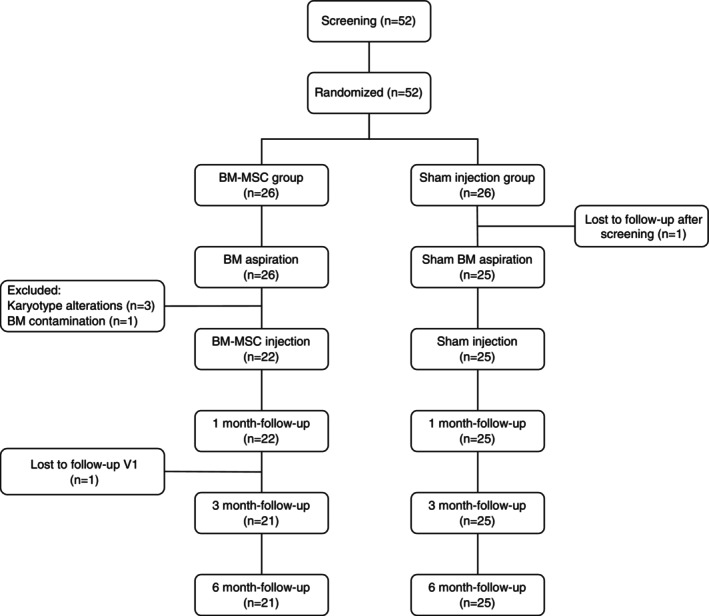
CONSORT flow diagram of the clinical trial.

A total of 52 patients were recruited from March 2021 to August 2024 and equally randomized to receive either intradiscal BM‐MSCs (*n* = 26) or a sham procedure (*n* = 26). In the BM‐MSC group, three patients were excluded due to karyotype alterations in the harvested cells and one additional subject was excluded due to BM contamination. Furthermore, two additional patients (*n* = 1 in the BM‐MSC group and *n* = 1 in the sham group) were lost to follow‐up. Eventually, 21 patients in the BM‐MSC group and 25 patients in the sham group completed the 6‐month follow‐up (Figure 1). Three‐month follow‐up MRI was missing in two patients in the BM‐MSC group and six patients in the sham group. At 6 months, follow‐up MRI was missing in three BM‐MSC patients and two sham‐treated subjects. Included patients had a mean age of 43.6 ± 11.1 years, were predominantly male (67.3%), and reported a baseline LBP severity of 57.7 ± 22.4 points according to the VAS. The complete demographic characteristics of the included patients are summarized in Table 1. No significant intergroup difference emerged following randomization, including the number of IVD levels and their degree of IDD as per the modified Pfirrmann grading.

**TABLE 1:** Baseline demographic and clinical characteristics of included patients.

Variable	BM‐MSC (*n* = 26)	Sham (*n* = 26)	*p*
Age, years (mean ± SD)	46.2 ± 10.6	41.1 ± 11.1	0.06
Sex (M/F)	16/10	19/7	0.38
Education level, *n* (%)			
Secondary education	7 (26.9)	2 (8.3)	0.12
Bachelor's degree	7 (26.9)	10 (41.7)	
Master's degree	9 (34.6)	5 (20.8)	
Postgraduate degree	3 (11.5)	7 (29.2)	
Alcohol consumption (drink/week, mean ± SD)	1.0 ± 1.6	0.3 ± 0.8	0.18
Smoking status, *n* (%)			
Never smoked	20 (76.9)	23 (92.0)	0.30
Ex‐smoker	1 (3.8)	0 (0.0)	
Current smoker	5 (19.2)	2 (8.0)	
Baseline VAS LBP (mean ± SD)	54.5 ± 22.6	60.8 ± 22.3	0.30
Baseline ODI (mean ± SD)	26.7 ± 15.3	28.9 ± 13.6	0.82
Baseline SF‐36 PCS (mean ± SD)	38.7 ± 9.2	37.0 ± 7.3	0.54
Baseline SF‐36 MCS (mean ± SD)	43.6 ± 11.4	43.0 ± 11.2	0.88
Baseline WAI (mean ± SD)	29.9 ± 8.3	30.5 ± 8.6	0.87
Number of levels	62	50	0.17
L1–L2	2	0	
L2–L3	7	4	
L3–L4	12	7	
L4–L5	19	19	
L5–S1	22	20	
Modified Pfirrmann grade			0.77
Grade 3	5	3	
Grade 4	15	12	
Grade 5	16	9	
Grade 6	14	12	
Grade 7	12	14	

Abbreviations: MCS = Mental Component Scale; ODI = Oswestry Disability Index; PCS = Physical Component Scale; SD = standard deviation; SF‐36 = Short Form‐36; VAS = visual analogue scale; WAI = Work Ability Index.

The average final cell dose was 14.8 ± 0.4 × 10^6^ BM‐MSCs per disc. Among the 22 treated patients, 20 received BM‐MSC injections in the number of IVDs initially planned (3 discs in 12 patients, 2 discs in 7 patients, and 1 disc in 1 patient). In two patients, the number of treated discs had to be reduced (from three to two in one case and from three to one in the other) due to insufficient cell yield. In these patients, a full 2 mL of injectate could be successfully delivered in all cases. All product batches met the predefined specifications for identity (%CD73^+^: 99.9% ± 0.2%, %CD90^+^: 99.8% ± 0.3%, %CD105^+^:99.5% ± 0.6%), contaminants (%CD45^+^:1.6% ± 0.9%), viability (propidium iodide‐negative cells: 96.7% ± 2.6%), and colony‐forming ability (CFU‐F: 44.3 ± 14.7 per each T25 flask). All administered products were tested negative for endotoxins, micro‐organisms, Mycoplasma, and exhibited normal karyotypes.

### 3.1 Clinical Outcomes

**FIGURE 2:**
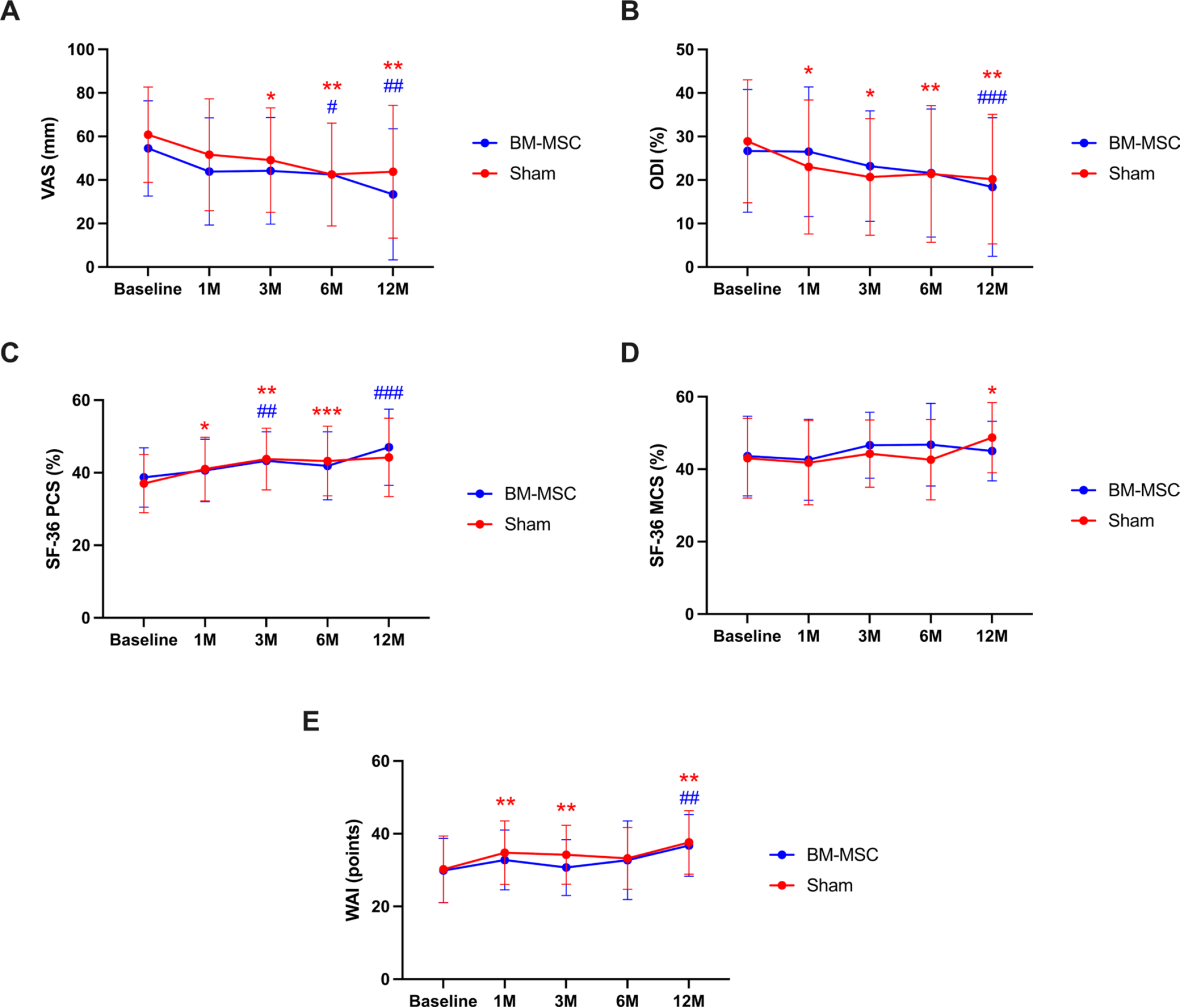
Evolution of LBP intensity (A), disability (B), quality of life according to the PCS (C) and MCS (D) scales, and work ability (F) throughout the study. BM‐MSC = bone marrow‐derived mesenchymal stroma cell; MCS = Mental Component Scale; ODI = Oswestry Disability Index; PCS = Physical Component Scale; SF‐36 = Short Form‐36; VAS = visual analogue scale; WAI = Work Ability Index. *p < 0.05, **p < 0.01, ***p < 0.001 in the sham group compared to baseline. ^#^ *p* < 0.05, ^##^ *p* < 0.01 in the BM‐MSC group compared to baseline.

Compared to baseline (sham: 60.8 ± 21.9; BM‐MSC: 54.5 ± 21.9), VAS scores (Figure 2A) showed a progressive reduction in LBP intensity in both groups. Significant improvements were observed at 3 months in the sham group (43.9 ± 24.6, *p* < 0.040) and at 6 months in both groups (BM‐MSC: 42.5 ± 23.6, *p* < 0.020; sham: 42.5 ± 23.6, *p* = 0.0002). ODI scores (Figure 2B) revealed no significant improvements in the BM‐MSC group, whereas the sham group demonstrated better scores at all timepoints (baseline: 28.9 ± 14.1; 1 month: 23.0 ± 15.4, *p* = 0.040; 3 months: 20.7 ± 13.4, *p* = 0.001; 6 months: 21.4 ± 15.7, *p* = 0.020). For quality of life, SF‐36 Physical Component Scale (PCS) scores (Figure 2C) were consistently higher in the sham group at all timepoints (baseline: 37.0 ± 8.0; 1 month: 41.0 ± 8.8; 3 months: 43.8 ± 8.5, *p* < 0.0001; 6 months: 43.2 ± 9.6, *p* = 0.0005), whereas the BM‐MSC group showed significant improvement only at 3 months compared to baseline (38.7 ± 8.2 vs. 43.3 ± 8.0, *p* = 0.003). In contrast, no significant differences were observed in Mental Component Scale (MCS) scores (Figure 2D) in either group. Similarly, WAI scores (Figure 2E) did not significantly improve in the BM‐MSC group, while the sham group exhibited significant improvements at 1 month (34.8 ± 8.7, *p* = 0.020) and 3 months (34.2 ± 8.1, *p* = 0.040) compared to baseline (30.2 ± 9.2). PROM values are shown in Table 2.

**TABLE 2:** LBP intensity, disability, quality of life, and work ability changes over the study course.

Score	Timepoint (month)	BM‐MSC				Sham		
		Mean (95% CI)	Difference from T0, mean (95% CI)	*p* (within group)	Mean (95% CI)	Difference from T0, mean (95% CI)	*p* (within group)	*p* (between groups)
VAS	0	54.5 (45.7; 63.4)			60.8 (51.9; 69.6)			
	1	43.9 (33.0; 54.8)	−10.6 (−22.1; 0.8)	0.070	51.6 (41.0; 62.2)	−9.0 (−20.3; 2.0)	0.110	0.850
	3	44.2 (33.1; 55.4)	−10.3 (−22.3; 1.7)	0.090	49.1 (39.2; 59.0)	−11.6 (−22.5; −0.8)	**0.040**	0.870
	6	42.5 (31.8; 53.3)	−12.0 (−22.3; −1.7)	**0.020**.	42.5 (32.8; 52.3)	−18.2 (−27.4; −9.0)	**0.0002**	0.370
ODI	0	26.7 (21.0; 32.4)			28.9 (23.2; 34.6)			
	1	26.5 (19.9; 33.1)	−0.2 (−6.0; 5.6)	0.940	23.0 (16.7; 29.4)	−5.9 (−11.5; −0.3)	**0.040**	0.160
	3	23.2 (17.4; 29.0)	−3.6 (−8.6; 1.5)	0.160	20.7 (15.1; 26.2)	−8.3 (−13.0; −3.5)	**0.001**	0.180
	6	21.6 (14.5; 28.8)	−5.1 (−11.8; 1.6)	0.140	21.4 (14.9; 27.9)	−7.5 (−13.5; −1.5)	**0.020**	0.590
SF‐36 PCS	0	38.7 (35.4; 42.0)			37.0 (33.7; 40.2)			
	1	40.6 (36.8; 44.4)	1.9 (−1.7; 5.4)	0.290	41.0 (37.3; 44.6)	4.0 (0.6; 7.4)	**0.020**	0.390
	3	43.3 (39.6; 46.9)	4.6 (1.6; 7.5)	**0.003**	43.8 (40.3; 47.3)	6.9 (4.1; 9.6)	**< 0.0001**	0.250
	6	41.9 (37.6; 46.2)	3.2 (−0.5; 7.0)	0.090	43.2 (39.2; 47.1)	6.2 (2.9; 9.6)	**0.0005**	0.240
SF36 MCS	0	43.6 (39.1; 48.0)			43.0 (38.5; 47.4)			
	1	42.6 (37.6; 47.5)	−1.0 (−5.7; 3.7)	0.670	41.8 (37.0; 46.6)	−1.2 (−5.7; 3.4)	0.610	0.960
	3	46.6 (42.5; 50.8)	3.1 (−1.9; 8.0)	0.220	44.3 (40.5; 48.2)	1.4 (−3.3; 6.0)	0.560	0.610
	6	46.8 (41.6; 52.0)	3.2 (−3.1; 9.6)	0.310	42.6 (38.0; 47.2)	−0.4 (−6.3; 5.5)	0.900	0.410
WAI	0	29.9 (26.4; 33.5)			30.2 (26.5; 33.9)			
	1	32.8 (29.1; 36.4)	2.8 (−0.9; 6.6)	0.140	34.8 (31.2; 38.4)	4.6 (0.8; 8.3)	**0.020**	0.520
	3	30.7 (27.2; 34.2)	0.8 (−3.1; 4.7)	0.690	34.2 (30.9; 37.6)	4.0 (0.2; 7.8)	**0.040**	0.240
	6	32.7 (28.8; 36.6)	2.8 (−1.6; 7.2)	0.210	33.2 (29.7; 36.7)	3.0 (−1.1; 7.1)	0.150	0.940

*Note:* Bold values represent statistically significant results.

Abbreviations: BM‐MSC = bone marrow‐derived mesenchymal stromal cells; CI = confidence interval; LBP = low back pain; MCS = Mental Component Scale; ODI = Oswestry Disability Index; PCS = Physical Component Scale; SF‐36 = Short Form‐36; VAS = visual analogue scale; WAI = Work Ability Index.

### 3.2 MRI Outcomes

**FIGURE 3:**
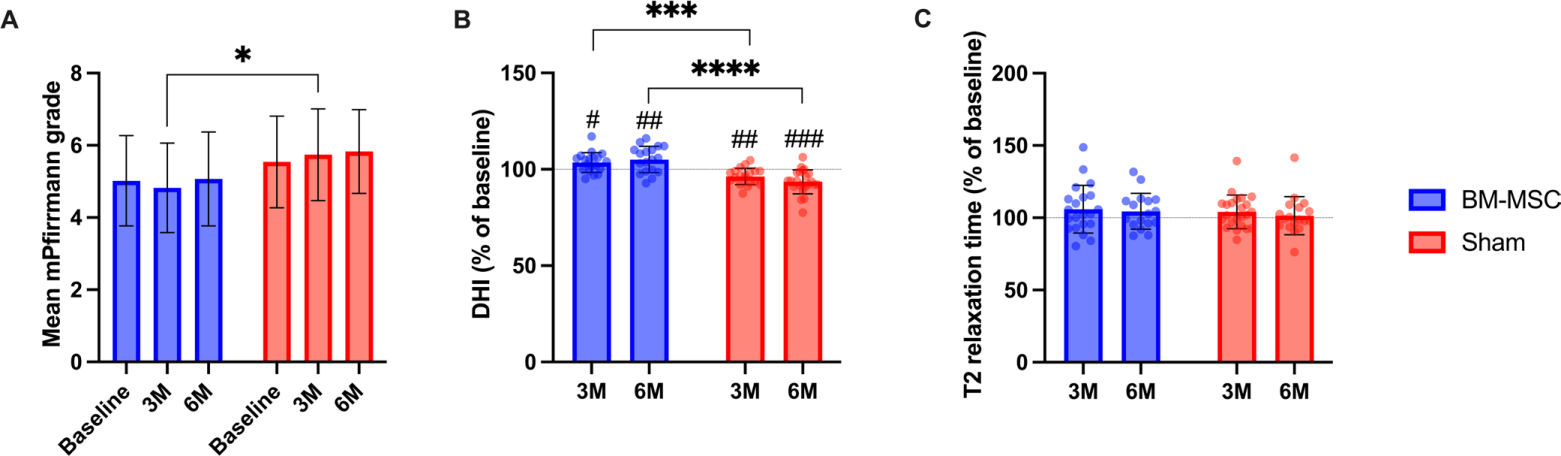
MRI analyses of mean modified Pfirrmann scores (A), DHI (B) and T2 relaxation times in the study groups. DHI and T2 relaxation time have been calculated as percent change compared to baseline. *p < 0.05, ****p < 0.0001 between groups. ^#^ *p* < 0.05, ^##^ *p* < 0.01, ^###^ *p* < 0.001 compared to baseline. BM‐MSC = bone marrow‐derived stromal cell; DHI = disc height index.

Starting from a baseline of 5.02 ± 1.25, the mean modified Pfirrmann grade of treated discs significantly decreased at 3 months compared to the sham group (4.82 ± 1.24, *p* = 0.024) but reached baseline levels by 6 months (Figure 3A). In contrast, no significant change was observed in the sham group. In the BM‐MSC group, DHI (Figure 3B) significantly increased at 3 months (103.5 ± 5.1%, *p* = 0.023 vs. baseline) and 6 months (105.1 ± 6.8%, *p* = 0.017 vs. baseline), with a significant difference between the two timepoints (*p* < 0.0001). Conversely, in the sham group, DHI significantly decreased compared to baseline at 3 months (96.3 ± 4.3%, *p* = 0.004) and 6 months (93.5 ± 6.2%, *p* = 0.0001), with a significant difference between timepoints (*p* < 0.0001). T2 mapping of treated IVDs (Figure 3C) demonstrated a slight improvement in signal relaxation times in the BM‐MSC group (104.5 ± 12.5%) compared to the sham group (101.4 ± 13.2%) at 6 months. MRI outcomes have been summarized in Table 3.

**TABLE 3:** Summary of MRI outcomes.

Outcome	BM‐MSC	Sham group	*p*
Modified Pfirrmann grade (mean ± SD)			
Baseline	5.02 ± 1.25	5.54 ± 1.27	0.162
3 months	4.82 ± 1.24	5.74 ± 1.27	0.025
6 months	5.07 ± 1.30	5.83 ± 1.13	0.551
DHI percent change (mean ± SD, %)			
Baseline	100.00 ± 0.00	100.00 ± 0.00	> 0.999
3 months	103.54 ± 5.14^#^	96.30 ± 4.28^##^	0.0002
6 months	105.10 ± 6.81^##^	93.50 ± 6.16^###^	< 0.0001
T2 relaxation time percent change (mean ± SD, %)			
Baseline	100.00 ± 0.00	100.00 ± 0.00	> 0.999
3 months	105.9 ± 16.5	104.1 ± 11.6	0.837
6 months	104.5 ± 12.5	101.4 ± 13.2	0.573

Abbreviations: BM‐MSC = bone marrow‐derived mesenchymal stromal cells; DHI = disc height index; SD = standard deviation.

^#^ *p* < 0.05.

^##^ *p* < 0.01.

^###^ *p* < 0.001 compared to baseline.

### 3.3 Safety Outcomes

In the BM‐MSC group, two TEAEs were observed: one case of transient worsening of LBP following the intradiscal injection, and one case of a cutaneous rash likely resulting from a reaction to the albumin in the cell suspension. Both events resolved promptly with medication and did not lead to further complications. The incidence of SAEs was similar between the groups (BM‐MSC: *n* = 4; sham: *n* = 3). One patient in the BM‐MSC group reported LBP worsening before the intradiscal injection, which rapidly improved following a short course of nonsteroidal anti‐inflammatory drugs. One subject in the sham group experienced a disc sequestration that became clinically evident at 6 months, resulting in study discontinuation; however, as the target disc was not punctured during the procedure, this event was not attributed to the study intervention. No cases of bleeding, infection, or death occurred. A summary of all AEs is provided in Table 4.

**TABLE 4:** Summary of adverse events observed during the first 6 months of follow‐up.

Category of SAE	BM‐MSCs	Sham
Subjects with any TEAE	2	0
Postprocedural LBP	1	0
Allergic cutaneous rash	1	0
*Subjects with any SAE*	4	3
Musculoskeletal disorders		
LDH requiring surgical decompression	0	1
LBP worsening	1	0
Gynecological disorders		
Hysterectomy with bilateral salpingo‐oophorectomy due to a benign tumor	1	0
Vascular disorders		
DVT	1	0
Renal disorders		
Renal colic with ureteral stent placement	0	1
Other		
Car accident	1	0
Insect sting with lower limb swelling	0	1

Abbreviations: DVT = deep venous thrombosis; LBP = low back pain; SAE = serious adverse event; TEAE = treatment‐emergent adverse event.

## 4 Discussion

This phase IIB randomized controlled trial represents, to our knowledge, the first investigation of autologous BM‐MSC therapy for chronic LBP due to multilevel moderate‐to‐advanced IDD. Our preliminary 6‐month results demonstrate that intradiscal injection of autologous BM‐MSCs is safe and possibly associated with slight structural improvements. However, these regenerative changes did not translate into a clear clinical advantage over the sham procedure in terms of pain, disability, and quality‐of‐life improvements.

The radiological findings suggest that BM‐MSC therapy may exert a modest regenerative effect on degenerated IVDs. The significant increase in DHI observed at both 3 and 6 months suggests that MSCs could potentially promote extracellular matrix synthesis and tissue hydration, leading to improved disc height. On the other hand, T2 mapping revealed only a slight, non‐significant increase in disc hydration in the BM‐MSC group compared to the sham group. These observations align with preclinical studies that have highlighted the capacity of MSCs to differentiate into NP‐like cells and secrete trophic factors that promote anabolic repair processes [9]. However, whether these mechanisms occur to a meaningful extent in the context of human degenerative IVDs remains to be fully elucidated. Aside from a slight improvement in modified Pfirrmann grading at 3 months, there were no significant differences between the groups. It is important to note that modified Pfirrmann grading may not reliably differentiate among moderate levels of IDD, whereas measurements of disc height have demonstrated higher intra‐ and inter‐rater reliability [20, 21]. According to a recent study, assuming a linear relationship between the number of injected cells and glycosaminoglycan (GAG) synthesis, administering a minimum of 10 × 10^6^ cells could restore approximately 65% of the GAG content found in a Grade 2 human IVD over a 10‐year period when applied to a Grade 3 IVD [22]. Given that our study employed a 1.5‐fold higher cell dose, the estimated timeframe for achieving similar regenerative outcomes is reduced to around 6 years. However, it remains uncertain whether improvements in tissue anabolism directly correlate with reductions in LBP [5]. Additionally, the observed increase in DHI may also be partially explained by the mechanical effect of injecting 2 mL of a water‐based solution into the IVD. Given that this volume represents approximately 10%–15% of the total disc volume (assuming a simplified disc geometry of 4 × 4 × 1 cm and full water content), it is plausible that the injected fluid volume transiently increases disc height in the absence of leakage. Moreover, introducing a high number of cells into the harsh microenvironment of degenerated discs, marked by competing nutrient demands, low pH, diminished glucose levels, and inflammation [2], could potentially worsen local conditions and challenge injected cell survival. These concerns are particularly relevant in patients with moderate‐to‐high grade IDD, characterized by an even more hostile microenvironment.

Clinical outcomes including VAS scores for LBP, ODI, and SF‐36 PCS improved in both treatment and sham groups, with some measures favoring the sham group and without any significant intergroup difference at any timepoint. This is in line with previous RCTs, where clinical improvements were consistently reported in both groups [11, 23, 24]. The discrepancy between structural regeneration and clinical symptomatology raises several considerations. First, it is well recognized that the relationship between disc morphology and pain is complex, with pain likely being modulated by multiple factors beyond the structural status of the IVD [25]. Second, the nature of the sham procedure itself might induce a placebo response, contributing to improvements in patient‐reported outcomes in the sham group [26]. While disc puncture itself may induce LBP and potentially worsen IDD, it is possible that the lower invasiveness of the intramuscular infiltration and the consequently reduced post‐procedural LBP compared to the BM‐MSC group would have further enhanced this discrepancy, as pointed out by Noriega et al. [24]. Additionally, the lack of specific approaches such as provocative discography to confirm which IVDs were the sources of each patient's LBP might explain the lack of significant clinical benefits in some cases. Finally, it is plausible that the regenerative effects of BM‐MSCs might require a longer time frame to manifest as clinically meaningful improvements. This aligns with the RCT by Amirdelfan et al. [12], who reported significantly improved VAS and ODI versus placebo only after 12 months. Therefore, extended follow‐up is warranted.

Our findings differ somewhat from earlier RCTs employing MSC‐based therapies for IDD. For instance, while the RESPINE study [11] observed a slightly higher percentage of responders in the cell‐treated group, it did not report significant intergroup differences in any of the PROMs over 2 years of follow‐up. Conversely, starting from 6 months, Amirdelfan and colleagues [12] demonstrated superior pain, disability, and quality‐of‐life outcomes in cell‐treated patients, especially with a higher dose (18 million cells) compared to a lower dose (6 million cells), relative to those treated with saline. A key feature of our study is the use of autologous BM‐MSCs, whereas previous investigations mainly utilized allogeneic products. Allogeneic MSCs offer advantages such as more convenient handling, lower production costs, and ease of use, with a generally favorable immune profile [13]. However, some studies have reported that allogeneic cell therapy may be compromised by the production of specific antibodies, potentially reducing MSC survival and resulting in a “hit and run” effect that may not sustain long‐term clinical improvements [27]. In this context, autologous cells could mitigate these issues through complete cytocompatibility, although the need for BM harvesting, cell expansion in dedicated facilities, and subsequent injection substantially increases treatment time, costs, and associated morbidity [28]. Compared to previous RCTs, we employed autologous BM‐MSCs in a population with multi‐level and more advanced degeneration, in contrast to many prior investigations that focused on single‐level or less severe cases. Patient selection, cell source, and methodological variations (e.g., cell dose, injection technique, and outcome measures) may all contribute to the observed differences [29]. It is conceivable that the regenerative capacity of MSCs might be more effective in earlier stages of degeneration or when used in conjunction with other therapeutic modalities (e.g., diverse analgesic or physical therapy regimens) or vehicles (e.g., hyaluronic acid) [30]. Similar to previous studies [31], the intradiscal injection was well‐tolerated, and no major AEs were attributed to the BM‐MSC therapy. This favorable safety profile supports the further exploration of MSC‐based strategies in the management of IDD, particularly given the limited treatment options for patients with chronic discogenic LBP.

This study has some limitations. The relatively small sample size and the short follow‐up period limit the generalizability of our findings and the ability to capture long‐term clinical benefits that might arise from structural disc regeneration, which may take months to years. Additionally, variations in cell yield, their intrinsic cellular characteristics, and the number of discs treated may have introduced heterogeneity that could affect outcome measures. Although the dropout rate was moderately low (*n* = 5, 9.6%), the uneven distribution of missing MRI data at 3 and 6 months may have skewed the results. Furthermore, the exclusion of 5 patients from the BM‐MSC group brought the group sample size below the minimum of 23 patients set by the power analysis, thus introducing additional bias. Future studies should aim to include larger cohorts and extend follow‐up durations to ascertain whether the early regenerative changes observed can eventually lead to sustained clinical improvements and to clarify the relationship between structural regeneration and symptomatic relief. Moreover, refining patient selection criteria, such as targeting individuals in the early stages of IDD or those with biochemically confirmed discogenic LBP, might further enhance the therapeutic impact of BM‐MSC injections. Given that patients with high‐grade, multilevel IDD are more likely to present with systemic comorbidities and advanced tissue deterioration, it is conceivable that this population might derive greater benefit from intradiscal injection of allogeneic MSCs derived from young, healthy donors, rather than from autologous MSCs. In this regard, the use of innovative quantitative MRI techniques such as MRI spectroscopy may help identify specific LBP phenotypes most likely to benefit from either approach.

## 5 Conclusion

Our preliminary data indicate that autologous BM‐MSC intradiscal injection is safe and possibly able to induce measurable structural improvements in patients with chronic LBP due to moderate‐to‐advanced multilevel IDD. However, the lack of a corresponding superior clinical benefit at 6 months highlights the complex interplay between disc regeneration and symptom relief in chronic LBP. These findings underline the need for longer‐term studies to fully determine the clinical impact of MSC‐induced structural regeneration and to optimize treatment protocols for patients with IDD.

## Conflicts of Interest

G.V. is an Editorial Board member of JOR Spine and coauthor of this article. He was excluded from editorial decision‐making related to the acceptance of this article for publication in the journal.

## Supporting information


**Data S1.** Supporting Information.
