# Supplementary material for: Intradiscal Mesenchymal Stromal Cell Therapy for the Treatment of Low Back Pain Due to Moderate‐to‐Advanced Multilevel Disc Degeneration: A Preliminary Report of a Double‐Blind, Phase IIB Randomized Clinical Trial (DREAM Study)
Source: JOR Spine. 2025 Jun 2;8(2):e70086. doi: 10.1002/jsp2.70086 (PMC12129703; doi:10.1002/jsp2.70086)
Supplement: Supplementary file 1 — Data S1. Supporting Information. [file JSP2-8-e70086-s001.pdf]

Clinical Study Protocol DREAM

Intervertebral Disc REgeneration mediated by Autologous Mesenchymal stem/stromal cells intradiscal injection: a phase IIB randomized clinical trial

Investigational Product:	**Autologous BM-MSC**
Date:	October 21th 2019
Development Phase:	2B
Brief title:	**Efficacy of intradiscal injection of autologous BM-MSC in subjects with chronic LBP due to multilevel lumbar IDD**
EudraCT number:	2019-002749-40
Sponsor reference code:	DREAM (GR – 2018-12367168)
Investigators:	Dr. Vadalà Gianluca
Sponsor:	Università Campus Bio Medico di Roma (UCBM)
Coordinating Contact:	g.vadala@gmail.com

This study will be conducted in compliance with the protocol, Good Clinical Pratice and all other applicable regulatory requirements, including the archiving of essential documents.

Table of content

A. Administrative Structure 6

B. Signature Sheets 9

C. Synopsis 11

D. Abbreviations 14

1.Introduction 16

1.1 Background 16
1.1.1. Epidemiology and pathogenesis of low back pain (LBP) 16
1.1.2. Diagnostic of lumbar IDD and current therapies 16
1.1.3. Innovative treatment of lumbar IDD 17
1.1.4. Mesenchymal stromal stem cell (MSC) 18
1.1.5. Regenerative therapy based on autologous MSC 19
1.2 Clinical trial rationale 20
1.3 Justification of the route administration, dosage and treatment period 22
1.4 Risks 23
1.5 Benefits 23
1.6 Risk-benefit assessment 23
1.7 Risk minimisation plan 24
2. Study design and endpoints 25

2.1 Study design 25

2.2 Randomization 26

2.3 Objectives 26

2.3.1 Primary objective: 26

2.3.2 Secondary objectives: 26

2.4 Endpoints 26

2.4.1 Primary Endpoint: 26

2.4.2 Secondary Endpoint: 27

3. Selection of Subjects/population(s) and patient follow up 27
3.1 Inclusion criteria 27
3.2 Exclusion criteria........ 28
3.3 Assignment of intervention and blinding 29
3.4 Patient follow up/Monitoring........ 30
3.5 Withdrawal of the clinical trial 30
3.6 Lost to follow up........ 30
3.7 Follow up after discontinuation 31
3.8 Replacement of patients........ 31
3.9 Follow up after termination 31
4. Measurement of outcomes 31
4.1 Primary Outcome Measures: 31
4.2 Secondary Outcome Measures:........ 31
5. Presentation of the Investigational Medicinal Product........ 34
5.1 Obtaining, purifying and expanding MSC......... 34
5.2 Methodology for obtaining bone marrow autologous 34
5.3 Packaging and conditioning for shipping of final product. 35
5.4 IMP labelling, storage and handling........ 35
5.5 Application of the cells 35
5.6 Application of sham procedure........ 36
5.7 Post-treatment care 36
5.8 Possible complications of this therapy. 37
6. Study procedures and visits........ 37
6.1 Schedule of assessment........ 37
6.2 Visits during the course of the trial........ 39
6.2.1 Pre-Screening visit........ 39
6.2.2 Screening visit........ 39
6.2.3 Inclusion visit and randomization 40
6.2.4 BM-MSC Withdrawall........ 40
6.2.5 Visit 0 (Day 0): Visit baseline and treatment 41
6.2.6 Visit 1: Month 1 42

6.2.7 Visit 2: Month 3 42
6.2.8 Visit 3: Month 6 43
6.2.9 Visit 4: Month 12 43
6.2.10 Visit 5: Month 24 44
6.3 Unscheduled visits 44
6.4 Concomitant therapy and Rescue therapy 44
6.5 Contraceptive methods 45
7. Statistic analysis planning and power calculation 46
7.1 Statistic analysis 47
7.2 Sensitivity analysis 47
7.3 Safety analysis 47
7.4 Handling of protocol deviations 47
7.5 Handling of dropouts or missing data 48
8. Assessment of Safety and Tolerability 48
8.1 Safety parameters 48
8.2 Definition 48
8.3 Expected adverse reactions 50
8.4 Unblinding procedure 52
8.5 Investigator responsibilities 52
8.6 Sponsor responsibilities 54
9 Data handling and record keeping 56
9.1 Investigator site file (ISF) 56
9.2 Trial Master file (TMF) 57
9.3 Obligation to archive (Principal investigators and sponsor) 57
9.4 Adherence to the clinical trial protocol 57
10 Data collection and data management 57
11 Quality control and quality assurance 58
11.1 Control of data consistency 58
11.2 On-site quality control 58
12 Monitoring, audits and inspections 59

12.1 Monitoring 59

12.2 Audit and Inspection 59

13 Informed Consent, Ethical Review, and Regulatory Considerations 60

13.1 Informed consent 60

13.2 Ethical review/Ccompetent authority approval 60

13.3 Regulatory Considerations 61

13.3.1 Responsibilities of the sponsor and investigators 61

13.3.2 Responsibilities of the Investigators 61

13.3.3 Patient confidentiality 62

13.3.4 Good Clinical Practice 63

13.3.5 Amendments to the clinical trial protocol 63

13.3.6 Declaration of End of trial 63

13.3.7 Final Report Signature 63

13.3.8 Financing and Insurance 64

13.3.9 Investigator Information 64

14 Clinical trial registry 64

15 Publication policy 64

16 Sponsor, coordinating centre(s) and committees 64

17. References: 65

This study will be conducted in compliance with the protocol, Good Clinical Practice and all other applicable regulatory requirements, including the archiving of essential documents.

A. Administrative Structure

Sponsor

University Campus Bio-Medico of Rome

Research Unit of Orthopedic and Trauma Surgery

Via Alvaro del Portillo, 200

00128 Roma

ITALY

Coordinating Investigator:

Dr. Vadalà Gianluca

Research Unit of Orthopedic and Trauma Surgery

University Campus Bio-Medico of Rome

Via Alvaro del Portillo, 200

00128 Rome, Italy

E-mail: g.vadala@gmail.com

Tel: +39 06 225411

Fax: +39 06 22541456

Cell engineering Units:

Dr. Tiziana Montemurro

Institution: CF Fondazione IRCCS Cà Granda Ospedale Policlinico Milano

Adress: Via Francesco Sforza 28

City: 20122 Milan

Country: Italy

Phone: +39 02 55034057

Fax: +39 02 55034057

Email: Tiziana.montemurro@policlinico.mi.it

Pharmacovigilance:

Dr. Andrea Di Mattia

Institution: Pharmacy – University Campus Bio-Medico of Rome

Adress: Via Alvaro del Portillo 200

City: 00128 Rome

Country: Italy

Phone: +39 06 225411157

Fax: +39 06 225411157

Email: a.dimattia@unicampus.it

Biostatistician:

Dr. Cinzia Leuter

Institution: University Campus Bio-Medico of Rome

Adress: Via Alvaro del Portillo 200

City: 00128 Rome

Country: Italy

Phone: +39 06 22541 9187

Fax: +39 06 22541 9187

Email: c.leuert@unicampus.it

Data Management:

Dr. Giuseppina Di Giacomo

Dr. Claudia Cicione

Dr. Francesca Cannata

Institution: University Campus Bio-Medico of Rome

Adress: Via Alvaro del Portillo 200

City: 00128 Rome

Country: Italy

Phone: +39 06 225419142

Fax: +39 06 225419142

Email: g.digiacomo@unicampus.it

claudia.cicione.rm@gmail.com

f.cannata@unicampus.it

B. Signature Sheets

SPONSOR PROTOCOL APPROVAL SIGNATURES

Name: Dr. Rossi Andrea

Title: Legal Representative of University Campus Bio Medico of Rome

Place, Date, Signature:
Rome,

Università Campus Bio-Medico di Roma
Il D[illegible]ettore Generale
D[illegible] [illegible]ndrea R[illegible]

COORDINATING INVESTIGATOR AGREEMENT AND SIGNATURE

The signature below constitutes approval of this protocol by the signatory and provides the necessary assurances that this study will be conducted according to all stipulations of the protocol and in accordance with ICH-GCP, the Declaration of Helsinki and local regulatory requirements.

Coordinating Investigator,

Name: Dr Gianluca Vadalà

21/10/19

Date

DOTT. GIANLUCA VADALÀ
CAMPUS BIO - MEDICO U.O.C ORTOPEDIA
9150915113601684

Signature:

Institution: Research Unit of Orthopedic and Trauma Surgery

University Campus Bio-Medico of Rome

Adress: Via Alvaro del Portillo 200

City: Rome

Country: Italy

Phone: +39 06-225411

Fax: +39 06-22541456

Email: g.vadala@unicampus.it

C. Synopsis

**Title**	**Intervertebral disc regeneration mediated by autologous mesenchymal stem/stromal cells intradiscal injection: a phase IIB randomized clinical trial - DREAM trial**
**Study type:**	Phase II B study
**Study design**	DREAM is a phase II B efficacy monocenter, prospective, randomized, controlled double blinded trial, comparing intra-discal autologous adult BM-MSC therapy and sham treated controls in subjects with chronic LBP (>6 months) due to lumbar multilevel IDD (max. 3 levels) unresponsive to conventional therapy.<br>Approximate duration of the recruitment period based on the number of patients available.<br>Duration of the recruitment period has been estimated at 12 months.
**Objectives**	***Primary objectives:***<br>• Evaluate the effectiveness of intradiscal injection of autologous BM-MSCs in reducing chronic LBP due to multilevel (max. 3 levels) lumbar IDD after 24 months of treatment in terms of pain relief (VAS), functionality (ODI) and quality of life (SF36).<br>***Secondary objectives:***<br>• Evaluate regenerative changes of the treated IVD.<br>• Assess modification of employment and work status between baseline and months 12 and 24.<br>• Assess safety and tolerability.<br>• Evaluate consumption of medications to relieve pain such as type and dose of analgesics.
**Subjects pupulation**	**Inclusion criteria**<br>• Age between 18 and 65 years.<br>• Signed informed consent.<br>• Symptomatic chronic LBP due to moderate IDD [modified Pfirrmann score 3-4 (Pfirrmann et al., 2001), Griffith score 3-7(Griffith et al., 2007)] at max.3 levels of the lumbar spine unresponsive to conservative treatment, physical and medical for at least 6 months. Physical treatment includes physiotherapy. Medical treatments includes NSAID, paracetamol, opioids and myorelaxant.<br>(For more details, see paragragh 1.1.4)<br>• Annulus fibrosus intact, demonstrated by MRI.<br>• Pain baseline > 40 mm on VAS (0- 100).<br>• NSAID washout of at least 2 days before screening.<br>• Painkillers washout of at least 24 hours before screening.<br>• For females of childbearing potential (see definitions in paragraph 6.5), a negative pregnancy test must be documented at Screening.<br>• Men and women should use effective contraception during treatment and for at least 24 months after BM-MSC discontinuation. The complete list of contraceptive methods is described in the patient information sheet and in the paragraph 6.5. As a precautionary measure, breast-feeding should be discontinued during treatment with BM-MSC and should not be restarted after discontinuation of BM-MSC.

	**Exclusion criteria**<br>• Congenital or acquired diseases leading to spine deformations that may upset cell application (scoliosis, isthmus lesion, sacralization and hemisacralization, degenetative spondilolisthesis).<br>• Spinal segmental instability assessed by dynamic X-Ray.<br>• Symptomatic facet joints syndrome on MRI (facet joints iperintensity and ipertrophy evaluated at coronal T2 weighted MRI).<br>• Prior to the screening visit, has received:<br>- Oral corticosteroid therapy within the previous 3 months, OR<br>• Intramuscular, intravenous or epidural corticosteroid therapy within the previous 3 months.Presence of a 4th level with symptomatic IDD (modified Pfirrmann score 3- 4, Griffith score 3-7) in the lumbar spine;<br>• Spinal canal stenosis (Schizas score > B).<br>• History of spinal infection.<br>• Lumbar disc herniation and sciatica.<br>• Endplate abnormality such as Schmorl's Nodes.<br>• Previous discal puncture or previous spine surgery.<br>• IDD with Modic II and III changes on MRI images.<br>• Patients not eligible to the intravertebral disc surgery.<br>• Patients who have the risk to undergo a surgery in the next 6 months.<br>• Patients with local infusion device/devices for corticosteroids.<br><br>• Obesity with body mass index (BMI in Kg/size in m^2) greater than 35 (obesity grade II).<br>• Participation in another clinical trial or treatment with another investigational product within 30 days prior to inclusion in the study.<br>• Abnormal blood tests: hepatic (alanine aminotransferase [ALT] and/or aspartate aminotransferase [AST] >1.5 × upper limit of normal [ULN]), renal, pancreatic or biliary disease, blood coagulation disorders, anemia or platelet count of <100 × 10^9/L.<br>• Pregnant or lactating women, or premenopausal women not using an acceptable form of birth control, are ineligible for inclusion. Contraception will be maintained during treatment and until the end of relevant systemic exposure. Additional pregnancy testing will be performed at the end of relevant systemic exposure. The patients will be required to use contraception from initial treatment administration until 24 months after the last dose of study drug.<br>• In each case of delayed menstrual period (over one month between menstruations) confirmation of absence of pregnancy is strongly recommended. The complete list of contraceptive methods is described in the patient information sheet and in the paragraph 6.5.<br>• Positive serology for following infection: Syphilis, HIV, Hepatitis B, or C.<br>• Contraindication to MRI assessed by the investigator.<br>• Intolerance or allergy to local anaesthesia.<br>• Any history of Cancer or immunodeficiency disease.<br>• Previous transplantation.

**Sample size and statistical method**	Sample size was calculated to have 80% power and 2 balanced groups and according to the literature data, a responder rate is expected at 12 months of the order of 30% in controls and 68% for MSC is a delta of 38% between the two groups. To highlight this difference while justifying a power of 80% and an alpha risk of 5% and within 1:1 ratio, taking into account 20% of inclusion failure, it is necessary to include 26 individuals per group a total of 52 subjects.
**Study duration**	39 months.<br>• Enrolment and therapy duration: 12 months.<br>• Follow-up: 24 months.

D. Abbreviations

Term	Abbreviation
ACBP	Adult Childbearing Potential
ADR	Adverse Drug Reaction
AE	Adverse Event
ATMP	Advanced Therapy medicinal product
BM-MSC	Bone Marrow autologous mesenchymal cells
CAs	Competent authorities
DDD	Degenerative Disc Disease
DSUR	Development Safety Update Report
ECs	Ethic Commitees
eCRF	electronical Case Report Form
FBS	Fetal Bovine Serum
GAG	Glycosaminoglycan
GMP	Good Manufacturing Practice
GVHD	Graft versus host disease
HLT	High Level Term
ICF	Informed Consent Form
IDD	Intervertebral Disc Degeneration
IVD	Intervertebral Disc
IMP	Investigational Medicinal Product
ISF	Investigator Master File
LBP	Low Back Pain
LPLV	Last Patient Last Visit
MAR	Missing At Random
MCAR	Missing Completely At Random
MCH	Major complex histocompatibility
MRI	Magnetic Resonance Imaging
MSC	Mesenchymal stromal stem cells

NSAID	Non Steroidal Anti Inflammatory Drug
NP	Nucleus Polposus
ODI	Oswestry disability index
PIL	Patient Information Leaflet
PT	Preferred Term
RCTs	Randomized Clinical Trials
RR	Relative Risk
SAE	Serious Adverse Event
SAR	Serious Adverse Reaction
SAP	Statistical Analysis Plan
SESAR	Suspected Expected Serious Adverse Reaction
SOC	System Organ Class
SmPC	Summary of Product Characteristics
SUSAR	Suspected Unexpected Serious Adverse Reaction
TMF	Trial Master File
VAS	Visual Analogic Scale
WOCBP	Women of childbearing potential

1. Introduction

1.1 Background

1.1.1. Epidemiology and pathogenesis of low back pain (LBP)

Low back pain (LBP) is the global leading cause of disability, and furthermore rates sixth in terms of overall disease burden, in both developed and developing countries (Maher et al., 2017). LPB is a condition of all ages, from children to elderly, affecting 60-70% of the global population during life, and ~700 million people (9.4%) each year (PopulationReferenceBureau; 2016 World population data sheet; Hoy D et al., 2014). LBP severely impacts patient health and Quality of Life, and being the leading cause of activity limitation and work absence, it causes enormous economic burden on healthcare, industry and government, by reducing productivity and increasing healthcare costs (Maher et al., 2017; Hoy et al., 2014). LBP prevalence increases with age, and with populations ageing worldwide, the societal and economic burden associated with LBP will increase substantially over coming years (Maher et al., 2017; Hoy et al., 2014).

Current LBP therapies are aimed at pain reduction, and do not provide restorative treatment. Such conservative strategies (e.g. painkillers and musculoskeletal rearrangement by manual and physiotherapy) rarely address the actual cause of LBP.

When all other strategies have failed, the last resort is invasive surgery, which demands extended rehabilitation and usually fails to provide full recovery and long-term pain alleviation (WHO, Background Paper 6.24 - Low back pain, 2013). Thus, there is a high unmet need for development of novel therapies to treat LBP.

In a healthy spine, intervertebral discs (IVDs) separate the vertebrae to provide complex spinal flexibility while supporting large spinal loads. Intervertebral disc degeneration (IDD) is widely recognized as a major contributor to LBP, responsible for at least 40% of LBP cases (Vadala et al., 2015). A key characteristic of IVD degeneration is loss of matrix integrity, thereby causing biomechanical functional failure. Herein, the resident cells play an essential role. During ageing, disc cells and disc-specific stem cells become exhausted, and thereby are unable to maintain matrix integrity. As such, to provide a curative treatment to LBP, restoring IVD function through IVD tissue regeneration and repair is required (Vadala et al., 2015).

1.1.2. Diagnostic of lumbar IDD and current therapies

Diagnosis of chronic LBP is made with reasonable certainty based on medical history and clinical examination. The patient suffers from persistent LBP more than 3 months, associated with morning stiffness limited in time and reduced functioning. The diagnosis is confirmed by imaging techniques and IDD can be

characterized by MRI showing evidence of a correlation between pain and the inflammatory aspect of discopathy, including loss of nucleus pulposus iperintensity at T2 waighted MRI, disc narrowing (Brinjikji et al., 2015).

Today, no therapy can restore intervertebral disc (IVD) function or provide long-term relief from symptomatic IDD. Conservative therapies (such as rest, behavioural, physical, manual or manipulative therapies, pharmacological agents, and lifestyle modifications) have not proven effective for IDD, but continue to be prescribed during acute episodes of back pain. Likewise, numerous minimally invasive interventional strategies have failed. These therapies targeted the neural or other biological elements of the disc via, for example, the intradiscal administration of steroids or electrothermal energy.

When these treatments fail, several types of surgery are performed to relieve pain and decrease disability. The most common interventions are spinal fusion (arthrodesis), disc surgery (discectomy and sequestrectomy) and ultimately artificial disc replacement. However, IDD surgery is controversial because of its side effects, disturbance of motion and other biomechanical consequences. Notably, surgery can accelerate the degenerative cascade at the pathological disc and at adjacent segments. Moreover, lumbar disc arthrodesis has failed to consistently demonstrate superior outcomes to non-surgical therapies, and has been associated with reoperation rates as high as 26% (Noriega et al., 2017).

1.1.3. Innovative treatment of lumbar IDD

New treatment strategies concentrate on treating IDD at an early stage. Stem cell research offers exciting possibilities, and advanced cell-based therapies are considered highly promising strategies in treating IVD degeneration and LBP (Vadala et al., 2016).

Encouraging results suggest that cell-based, regenerative therapies may provide the world first effective therapy for this common and debilitating disease. In previous phase I clinical trials, patients (#10) affected by LBP due to early IDD exhibited rapid and progressive improvement of functional indexes of 65% to 78% over 1 year after intradiscal administration of autologous bone marrow mesenchymal stromal/stem cells (BM-MSC) with not side effect reported (Orozco et al., 2011). In a second study, a pilot controlled randomized clinical trial (RCT) using allogeneic BM-MSC has been conduced in 24 patients (phase IIa) compared to a sham treatment. The procedure appeared to be safe, and no side effects were reported. The pain score and the Oswestry disability index (ODI) were reduced by about 50% at 6 months following the intervention, while the control, sham-treated patients showed no significant benefit (Noriega et al., 2017). In addition, magnetic resonance imaging (MRI) T2 relaxation measurements demonstrated a significant improvement of IVD signal, in the 12 patients treated with allogenic BM-MSC compared to sham control (Noriega et al., 2017).

MSC represent a promising opportunity, but only RCTs can determine whether it is a truly effective alternative to spine fusion or disc replacement. Potential

advantages of these treatments are preservation of normal surrounding anatomy, biomechanics, and motion. To accomplish this goal, we propose an ambitious RCT aimed at developing a clinically applicable treatment for IDD based on autologous BM-MSC. Indeed, according our knowledge, no RCTs have been performed using MSC from autologous source. Moreover, tissue regeneration evaluated using advanced MRI imaging technologies has not been evaluated in RCTs.

1.1.4. Mesenchymal stromal stem cell (MSC)

Mesenchymal stem cells, or stromal cells (MSC) are progenitor cells mainly isolated from adult bone marrow (BM), and adipose tissue. The last two localizations allow easier collection and therefore an extension of their use. They have several functions: synthesis of extracellular matrix, immune tolerance, development, anti-inflammation and fibrosis. MSC are defined by their functional abilities to differentiate and differ from hematopoietic stem cells by the expression of mesenchymal markers (CD105, CD70, CD90), while lacking expression of CD34, CD45, CD14 monocyte or markers of T or B cells, or the major histocompatibility class II (MHC II). MSC have a phenotypic heterogeneity with some multipotent properties and are the progenitors of multiple lineages including bone, cartilage, muscle or fat. MSC are currently being studied for tissue engineering applications, including bone and cartilage repair because of their potential to differentiate into different lineages such as chondrocytes, osteoblasts or adipocytes.

MSC have immunomodulatory and immunosuppressive properties and are involved in both the innate and the adaptive immunity. This immunosuppressive effect is mainly due to the secretion of soluble factors by MSC and by direct contact with immune cells. MSC acquire their immunosuppressive properties after exposure to an inflammatory environment. Some cytokines such as tumor necrosis factor alpha (TNF-α), interleukin 1 beta (IL-1-β) or interferon gamma (IFN-γ) are able to activate MSC.

In tissue repair, MSC do not seem to have a direct effect, but they stimulate the regenerative properties of resident cells. They have a paracrine effect by reducing the release of pro-inflammatory cytokines and by stimulating the secretion of antiinflammatory cytokines. MSC exert their regulatory role by forming a perivascular niche in close contact with endothelial cells and osteoblasts in bone marrow and in close relationship with the immune and hematopoietic stem cells. MSC operate in collaboration with endothelial factors (epidermal growth factor (EGF), vascular endothelial growth factor (VEGF), insulin-like growth factor 1 (IGF-1), stromal cellderived factor 1 (SDF-1), transforming growth factor (TGF), Angiopoietin 1) to improve their “homing” on damaged sites.

MSC represent a promising opportunity, but only high-quality randomized controlled trials, comparing it to “standard of care” (indometacina, aceclofenac, diclofenac combinations, sulindac, diclofenac, phenylbutazone, valdecoxib,

rofecoxib, celecoxib, etoricoxib, tolfenamic, tenoxicam, meloxicam, piroxicam, dexibuprofen, tiaprofenic acid, flurbiprofen, ketoprofen, naproxen, ibuprofen, nabumetone,Eperisone Hydrochloride, Thiocolchicoside, Cyclobenzaprine, Tizanidine Hydrochloride, Codeine (FDA Drug Safety Communication,2015)(Cartabellotta et al., 2017), can determine whether it is a truly effective alternative to spine fusion or disc replacement. Potential advantages of these treatments are preservation of normal surrounding anatomy, biomechanics, and motion. To accomplish this goal, we propose an ambitious clinical project aimed at developing a broadly available and clinically applicable treatment for IDD. This project will provide that rigorous, clinical proof and will direct future efforts in Italy and Europe.

1.1.5. Regenerative therapy based on autologous MSC

Regenerative stem cell therapies for a number of conditions are in the first phases of clinical trials. There are about 2,000 listed at clinicaltrials.gov (excluding blood stem cell applications). These first phases test only safety; nonetheless, positive signs are often seen. A recent stem cell trial for blindness (macular degeneration) partially restored the vision of two subjects (Orozco et al., 2011). Pre-clinical data shows numerous potential advantages of regenerative strategies for treatment, such as preservation of normal surrounding anatomy, biomechanics, and motion. Mesenchymal cell therapy has produced exciting results both *in vitro* and *in vivo*. Studies with MSC have been particularly promising (Blanco et al., 2010). In our previous *in vitro* study, the co-culture of human MSC with nucleus pulposus (NP) cells results in upregulation of both NP cell proliferation and activity and MSC differentiation towards the chondrogenic lineage (Vadalà et al., 2008). Recent research indicates that NP contains populations of MSC that are very similar to the MSC recovered from bone marrow (Peng et al., 2013; Hiyama et al., 2008). Beside chondrogenic potential, MSC have shown an anti-inflammatory effect through the release of IL1RA, TSG6, IDO, iNOS, and other anti-inflammatory molecules. MSC are able to inhibit T cell proliferation as well as monocyte activation. This anti-inflammatory effect is important in the benefit observed in IDDD where local inflammation is induced by cartilage degradation. Studies in animal models of disc degeneration have shown that MSC injected in the NP area not only survive for months but also proliferate in canine, porcine and rabbit models (Henriksson et al., 2009; Yang et al., 2010). In addition, transplanted MSC induced production of IVD extracellular matrix proteins, including proteoglycan, aggrecan, and types I and II collagens (Ankrum et al., 2014). Studies from literature were conducted on dogs which underwent a partial nucleotomy at 3 lumbar levels (L3-L4, L4-L5, and L5-L6); adjacent levels served as nonoperated controls. Assessments of the experimental discs plus the 2 adjacent untouched discs were made using MRI, radiography, histology, and biochemistry at 12 months. Disc repair was clearly demonstrated through histology and biochemical analysis: disc injected with MSC more closely resembled the healthy controls as evidenced in matrix translucency, compartmentalization of the annulus, and in cell density within the NP. Matrix analysis for Type-II collagen and aggrecan demonstrated evidence of a better regenerative stimulation to the disc provided by MSC when compared to

controls19c. Finally, these studies also reported that injection of MSC resulted in better preservation of the height and water content of discs (Serigano et al., 2010; Hiyama et al., 2008).

Further proofs of NP regeneration have been obtained by our group in another large size animal study (sheep) conducted by members of the consortium (UCBM). A partial nucleotomy model has been used to test autologous BM-MSC (produced by the Cell Factory) transplantation with cell dose escalation (low or high doses). We have delivered 100µl of the hyaluronan-based hydrogel with a suspension of low or high doses of BM-MSC, using the nucleotomy model without injection as control. We have shown that a high dose of autologous MSC ($1x10^7$ cell/ml) regenerates the NP IVD in this model with high cell engraftments up to one year after transplantation demonstrated by sequential MRI, gross anatomy evaluation and histology.

Moreover, X-Ray evaluation showed the maintenance of the disc height index (DHI) of the high dose MSC treated group compared to partial nucleotomy control [unpublished data].

More than 450 trials are ongoing in 2016 using allogeneic MSC in regenerative medicine worldwide, with a major focus on cardiovascular pathology, chronic inflammatory diseases, and musculoskeletal pathologies, based on allogeneic cells in most cases (Imran et al., 2015). We propose a large-scale double-blind controlled trial to validate IDDD therapy based on autologous BM-MSC.

1.2 Clinical trial rationale

We hypothesize that autologous BM-MSC intradiscal injection in patients affected by LBP due to moderate multilevel lumbar IDD will be able (1) to significantly reduce LBP and improve functional status compared to sham controls. Moreover, the treated IVD will (2) undergo regeneration in terms of MRI changes compared to the sham control group. We also hypothesize that the proposed cell therapy is (3) safe in the long term.

The PI has obtained significant preliminary data in the past 15 years of research activity developing a MSC based therapy for the treatment of IDD. A series of consecutive studies from life science to preclinical tests on large animal models with long follow up have been conducted in order to obtain the data necessary to move toward the clinical translation. *In vitro* long follow up have been conducted in order to obtain the data necessary to move toward the clinical translation. *In vitro* studies of the PI suggested the regenerative potential of MSC results from MSC and NP cells interactions that up-regulate extracellular matrix protein synthesis (Orozco et al., 2011; Blanco et al., 2010). This synergistic effect is due to a combination of MSC differentiation toward NP-like cells and the trophic effect of MSC upon NP cells (Vadalà et al., 2008). The PI also tested the feasibility of the approach in a small animal model (rabbit) with BM-MSC expanded in standard condition showed cell engraftment up to 6 months after transplantation (Blanco et al., 2010). Proof of efficacy has been obtained in a preclinical large animal model (ovine) of IDD (grade IV) developed by the PI (Peng et al., 2013). A dose escalation

study has been conduced to test BM-MSC transplantation within a nucloteomized disc. We have shown that a high dose of autologous BM-MSC (1x10^7 cell/ml) regenerates the NP in a preclinical model of IDD up to one year after transplantation compared to control group. (Fig. 1) [unpublished data].

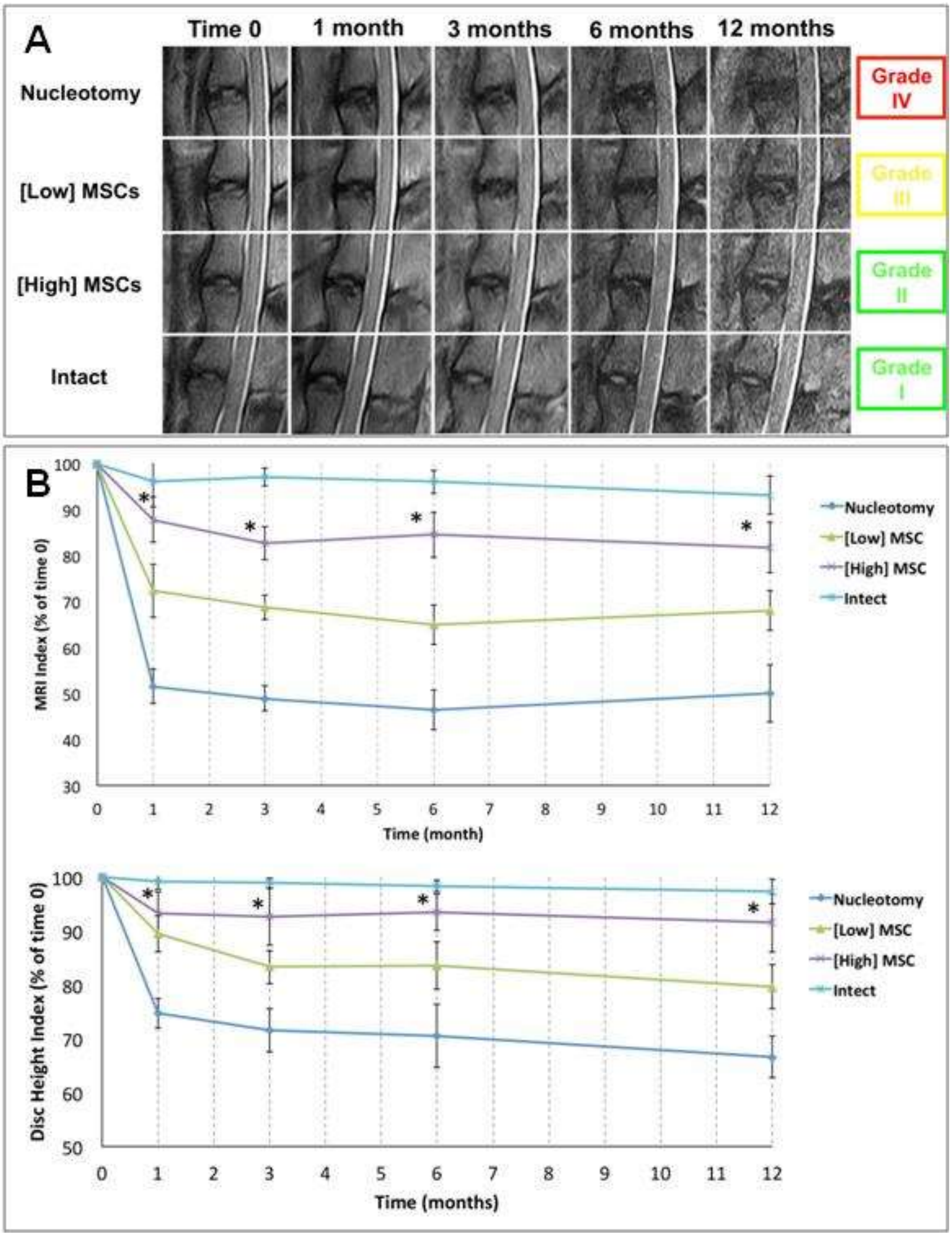

FIGURA 1A: L'intensità del segnale del tempo di rilassamento T2, ottenuta dalla RMN del NP e valutata seguendo il grado degenerative secondo Pfirrmann, ha mostrato che i dischi che hanno ricevuto l'iniezione di MSC ad alte dosi appariva di grado II, i dischi con basse dosi di MSC grado III e il gruppo non trattato è apparso come di grado V; **FIGURA 1B:** I dischi trattati con alte dosi di CF-BM-MSC hanno mostrato mantenimento dell'indice RMM rispetto al disco sano e un livello significativo rispetto al controllo negativo.

These recent results have been obtained thanks to a cooperative project coordinated by the PI with the cell factory(Young Investigator Grant of the Italian Ministry of Health, GR-2010-2318448). In the framework of this project Cell Facory have validated the manufacturing process and prepared the Investigational Medicinal Product Dossier (IMPD) containing the quality and non-clinical data on BM-MSC for IVD regeneration.

Indeed, Cell facotry is a reliable partner since it has been involved as GMP manufacturing center in several national and international clinical trials. In particular, in the orthopedic field, it provided BM-MSC for a multicentric European clinical trial for bone regeneration in non-union long bone fracture (NCT01842477). In this trial 200 million GMP expanded autologous BM-MSC, combined during surgery with bioceramics, safely obtained bone consolidation in 26 out of 28 patients (92%) with diaphyseal or metaphysodiaphyseal nonunions of femur, tibia or humerus. The PI hold also expertise in conducing RCTs in stem cell therapy. Indeed, the PI at UCBM is currently involved in a multicentric European RCT for disc regeneration. In this project, named RESPINE, allogenic BM-MSC are currently being tested for the treatment of LBP and disc regeneration. UCBM is the solo Italian center involved in the RCT.

1.3 Justification of the route administration, dosage and treatment period

Autologous BM-MSC will be administered via imaging control into the discs affected by IDD where they are expected to exert their therapeutic effects. As we mentioned above, no current standard treatments are available to influence the course of the disease and no drugs/devices brought significant proofs to limit the structural IDD. In the previous studies, a median dose of $15X10^6$ of MSC was injected with a good outcome and without side effects. Thus, we decided to use a dose of $15X10^6$ of autologous BM-MSC each disc in the active arm and a sham procedure in the control group without active product.

Several studies have reported that BM-MSC injection is safety, without adverse events [Orozco et al., 2013] [Vega et al., 2015]. Mild adverse reactions, such as pain, have been reported for both groups (sham and treated) without statistically significant differences.

The possibility to treat 3 different discs, each one with the selected dose ($15 X 10^6$), has been decided according to the results of a recent Phase I clinical study where $50 X 10^6$ Adipose derived stem cells were intrarticularly injected without adverse events [Pers et al., 2016].

Based on previous recommended papers, it seems the best option to check efficacy in chronic LBP is a period of more than 6 months. Thus, we retained as primary outcome an improvement of pain and functional status 12 months after treatment.

1.4 Risks

A large number of trials have risen rapidly investigating the efficacy in treating conditions such as type I and II diabetes, liver cirrhosis and regeneration, fistulas, cardiovascular disease (ClinicalTrials.gov Identifier NCT00999115), limb ischemia, amyotrophic lateral sclerosis and lipodystrophy (http://clinicaltrials.gov). More than 400 trials have been conducted with more than 1000 patients treated with MSC. Furthermore, BM-MSC are also under examination in clinical case studies for graft-versus-host disease immunosuppression, rheumatoid arthritis, Crohn's disease and ulcerous colitis, multiple sclerosis, soft tissue augmentation and bone tissue repair (Fang et al., 2006; Fang et al., 2007) with a clinical benefit, as well as appropriated tolerance. Clinical experience is available with both autologous and allogeneic BM-MSC administration.

- Systemic effects: The autologous nature of the study medication effectively excludes the risks of transmission of infectious agents, graft rejection, or GVHD (Graft versus host disease). The risk of systemic infection, bacteraemia, or sepsis due to contamination of the cell preparation is negligible. .
- Local effects: the complications are the same as in the discography, and rarely presented. The most common complaint is the exacerbation of pain for 1-2 weeks, which is usually solved by analgesia and muscle relaxants for a short period. The most serious complication is discitis, occurring in less than 0.1 % (Osti et al., 1990). Procedure related adverse events such as of infections, bleedings, nerve irritations and nerve injuries with its possible consequences paresthesia and paralysis are exceptional during the injection using the CT scan or fluoroscopic guidance, but patients will be informed.

1.5 Benefits

The objective of this clinical trial is to generate efficacy and tolerability profiles of a single injection of $15x10^6$ cell/ml each of autologous BM-MSC for each disc affected by IDD (up to 3 discs) versus sham procedure. The affected discs (max 3) are treated simultaneously once only, at the V0 visit and no subsequent doses are expected.

The potential of BM-MSC to lead to a disease-modifying therapeutic option for the treatment of this chronic and debilitating disease will be assessed by MRI after 6 months, 1 and 2 years.

1.6 Risk-benefit assessment

This study will progress beyond the current state of the art as it will be the one of the first clinical trial to reach significance in assessing autologous BM-MSC for the treatment of IDD. This study provides the first major step in determining subsequent clinical and commercial activity relating to stem cell therapy. It will definitively

provide robust in patient regenerative medicine research that either supports or refutes the potential of intradiscal injection of BM-MSC for the treatment of LBP due to IDD grade 3-4 modified Pfirrmann and grade 3-7 Griffith grading system at different levels (max. 3 levels). If successful, this will allow a new therapy to be taken to the next level of testing, taking the field closer to marketability and delivery, a key step that has eluded stem cell therapies to date.

Based on previous clinical experience with similar products reported in the literature, BM-MSC is deemed to be effective and safe in various diseases. Manufacturing of the study medication is performed according to GMP standards. The risk of local or systemic infection, bacteremia, or sepsis due to contamination of the cell preparation seems negligible. Pre-clinical *in vitro* and *in vivo* evaluations of the study medication did not show any hint of tumorigenic potential of the preparation or systemic migration of BM-MSC after intra-discal injection. There is a low risk of systemic AEs occurring after the end of the observation period, but the sample size of this clinical trial implies a very low probability of detecting rare events anyway. At last, procedure related adverse events such as of infections, bleedings, nerve irritations and nerve injuries with its possible consequences paresthesia and paralysis are very rare as the injection are performed using the fluoroscopy to guide the surgeon, but patients will be informed.

The benefit for patients with chronic LBP may be considerable, since BM-MSC might represent the first disease-modifying therapeutic option for this chronic and debilitating diseaseFurthermore, under certain pre-defined conditions, e.g. the occurrence of suspected unexpected serious adverse reactions (SUSARs), the Sponsor will suspend treatment of subjects until a decision whether to continue the clinical trial or not has been taken. In conclusion, subjects will be exposed to limited risks during their participation in this clinical trial.

Therefore, the risk-benefit ratio for this clinical trial is anticipated to be favourable and advocates its conduct in the selected group of subjects. Members of the a pilot trial demonstrating the feasibility and safety of the procedure has been already carried out. The currently proposed trial would be performed by experienced researchers who have already tested cell therapy materials and protocols with the following characteristics.

1.7 Risk minimisation plan:

The sham treatment in the control arm is not harmful to the patient: no disc injection, no placebo injection will be performed in the control patients. The patients with IDD are chronic patients, painful but with no vital risk or no significant risk of delaying effective therapy. The 6 months follow up with conventional therapy is not a reduction of chance to IDD cure as it is a slow irreversible disc degeneration.. Conventional treatment as physiotherapy or analgesic is allowed during the trial period (NSAID and oppioid as suggested by “standard of care” [Cartabellotta et al., 2017]). In case patients became painful and who develop “peripheral neurological deficit” they will undergo electromyography of the legs. In case the results obtained show pinched sciatic nerve, the investigator will decide to exclude the patients from

clinical trial and shift the patient to the appropriate standard of care (NSAID, steroid injection, rest, physiotherapy)[Cartabellotta et al., 2017]. In case of severe neuropathic pain (sciatica), the patient will be allowed to perform surgery, he will be considered as a failure. We enclose adequate risk minimization measures in the protocol avoiding the inclusion of patients who are not eligible to the intravertebral disc surgery or so painful that they have high probability to undergo surgery in the next 6 months period as well as patients with herniated discs.

2. Study design and endpoints

2.1 Study design

DREAM is a phase 2 b efficacy monocenter, prospective, **randomized, controlled double blinded trial**, comparing intra-discal autologous adult BM-MSC therapy and sham treated controls in subjects with chronic LBP (> 6 months) due to lumbar multilevel IDD (max. 3 levels) unresponsive to conventional therapy. Patients will be randomized in 2 arms of 26 patients and followed up for 24 months.

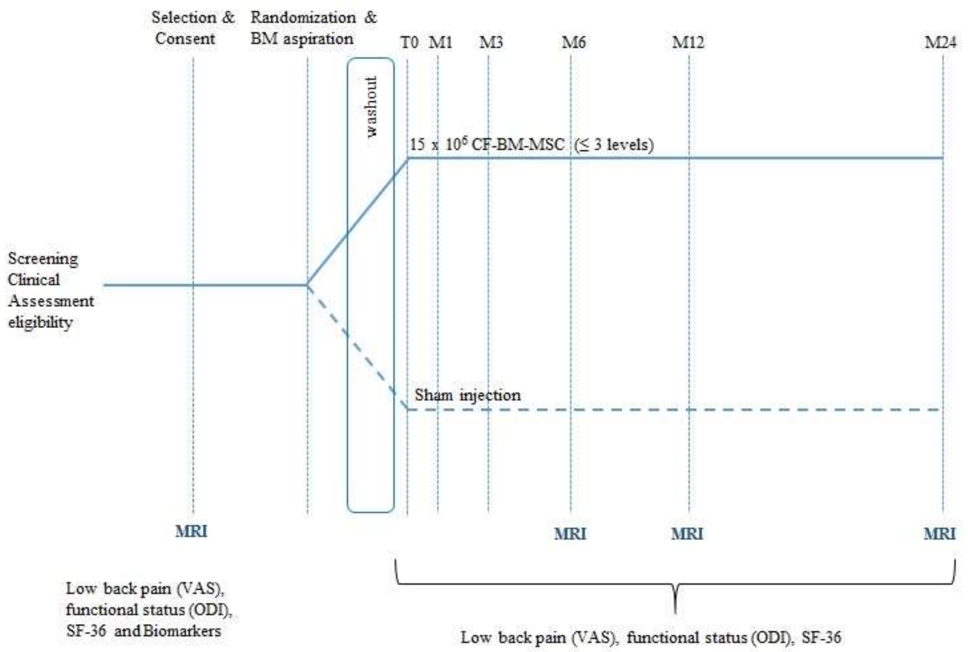

Figure 4 - Study design

DREAM will use autologous BM-MSC expanded according to validated and approved IMPD, using same cell process as in a previous assay authorized by EMA and performed by members of the consortium.
Bone marrow will be obtained from the patients after inclusion in the study and informed consent signature. The sham group will undergo percutaneous puncturing, without the needle gets to the iliac crest and without bone marrow withdrawal.

2.2 Randomization

The randomization will be centralized and stratified on the investigation center, and on the current attending to a rehabilitation programm at the inclusion visit. It is performed 24 hour-a-day online by the central randomization web-service.To randomize a patient, investigators have to login, enter the patient identification, additional checks and factor values. Since patients are assigned to the centre, the randomizing investigator is currently in the system. The patient identification must be unique within all randomizations performed. If everything is entered correctly, a summary of the patient data including the randomization result is shown. For double-blind trial the randomization result will be a treatment code. The Investigator in charge of injections will not have any discussion about treatment allocation with patients and clinical observers. The PI won't have access to the list of treatment codes used for blinding. Thereby will transmit the treatment code of the patient to each local pharmacy.

2.3 Objectives

2.3.1 Primary objective:

• Evaluate the effectiveness of intradiscal injection of autologous BM-MSCs in reducing chronic LBP due to multilevel (max. 3 levels) lumbar IDD after 24 months of treatment in terms of pain relief (VAS), functionality (ODI) and quality of life (SF36).

2.3.2 Secondary objectives:

- Evaluate regenerative changes of the treated IVD.
- Assess modification of employment and work status between baseline and months 12 and 24.
- Assess safety and tolerability.
- Evaluate consumption of medications to relieve pain such as type and dose of analgesics.

2.4 Endpoints

2.4.1 Primary Endpoint:

- To evaluate the efficacy of intradiscal injection of autologous BM-MSC using the visual analog scale (VAS) and functional status assessed by the Oswestry disability index (ODI) after 24 months of treatment, defining

responders in case of at least 40% improvement in VAS or ODI at month 24 compared to baseline.

2.4.2 Secondary Endpoint:

- To evaluate changes of the treated IVD induced by regenerative therapy by quantitative Magnetic Resonance Imaging (MRI) signal measurements in T2, T1spin/echo, and T1rho weighted images that allow a quantitative measure of the NP extracellular matrix.
- To evaluate modification of disability (ODI) and quality of life (SF-36 scores) considered as continuos measures, between baseline and 1, 3, 6, 12 and 24 months.

- Safety and tolerability will be evaluated by recording adverse events (AEs) and serious AEs (SAEs) throughout the study till 24 months. Number of participants with adverse events, the correlation of the AE to the IMP and their severity will be used as a measure of safety and tolerability.
- To evaluate consumption of medications to relieve pain such as type and dose of analgesics will be evaluated. Paracetamol (acetaminophen) and levels 2 analgesics will be assessed throughout the study at each visit.

3. Selection of Subjects/population(s) and patient follow up

This study will include patients affected by IDD in the lumbar spine and persistent LBP that do not respond to conservative treatment, physical and medical (NSAID or oppioids), lasting at least 6 months. A pain baseline value over 40/100 on visual analogue scale (VAS) shall be required.

3.1 Inclusion criteria

- Age between 18 and 65 years.
- Signed informed consent.
- Symptomatic chronic LBP due to moderate IDD [modified Pfirrmann score 3-4 (Pfirrmann et al., 2001), Griffith score 3-7(Griffith et al., 2007)] at max.3 levels of the lumbar spine unresponsive to conservative treatment, physical and medical for at least 6 months. Physical treatment includes physiotherapy. Medical treatments includes NSAID, paracetamol, opioids and myorelaxant. (For more details, see paragragh 1.1.4).
- Annulus fibrosus intact, demonstrated by MRI.
- Pain baseline > 40 mm on VAS (0- 100).NSAID washout of at least 2 days before screening.
- NSAID washout of at least 2 days before screening.

- Painkillers washout of at least 24 hours before screening.
- For females of childbearing potential (see definitions in paragraph 6.5), a negative pregnancy test must be documented at Screening.
- Men and women should use effective contraception during treatment and for at least 24 months after BM-MSC discontinuation. The complete list of contraceptive methods is described in the patient information sheet and in the paragraph 6.5. As a precautionary measure, breast-feeding should be discontinued during treatment with BM-MSC and should not be restarted after discontinuation of BM-MSC.

3.2 Exclusion criteria

- Congenital or acquired diseases leading to spine deformations that may upset cell application (scoliosis, isthmus lesion, sacralization and hemisacralization, degenetative spondilolisthesis).
- Spinal segmental instability assessed by dynamic X-Ray.
- Symptomatic facet joints syndrome on MRI (facet joints iperintensity and ipertrophy evaluated at coronal T2 weighted MRI).
- Prior to the screening visit, has received:
  - Oral corticosteroid therapy within the previous 3 months, OR
  - Intramuscular, intravenous or epidural corticosteroid therapy within the previous 3 months
- Presence of a 4th level with symptomatic IDD (modified Pfirrmann score 3-4, Griffith score 3-7) in the lumbar spine.
- Spinal canal stenosis (Schizas score > B).
- History of spinal infection.
- Lumbar disc herniation and sciatica.
- Endplate abnormality such as Schmorl’s Nodes.
- Previous discal puncture or previous spine surgery.
- IDD with Modic II and III changes on MRI images.
- Patients not eligible to the intravertebral disc surgery.
- Patients who have the risk to undergo a surgery in the next 6 months.
- Patients with local infusion device/devices for corticosteroids.

- Obesity with body mass index (BMI in Kg/size in m^2) greater than 35 (obesity grade II).

- Participation in another clinical trial or treatment with another investigational product within 30 days prior to inclusion in the study.
- Abnormal blood tests: hepatic (alanine aminotransferase [ALT] and/or aspartate aminotransferase [AST] >1.5 × upper limit of normal [ULN]), renal, pancreatic or biliary disease, blood coagulation disorders, anemia or platelet count of <100 × 10^9/L.
- Pregnant or lactating women, or premenopausal women not using an acceptable form of birth control, are ineligible for inclusion. Contraception will be maintained during treatment and until the end of relevant systemic exposure. Additional pregnancy testing will be performed at the end of relevant systemic exposure. The patients will be required to use contraception from initial treatment administration until 24 months after the last dose of study drug.

- In each case of delayed menstrual period (over one month between menstruations) confirmation of absence of pregnancy is strongly recommended. The complete list of contraceptive methods is described in the patient information sheet and in the paragraph 6.5. Positive serology for following infection: Syphilis, HIV, Hepatitis B, or C.
- Contraindication to MRI assessed by the investigator.
- Intolerance or allergy to local anaesthesia.
- Any history of Cancer or immunodeficiency disease.
- Previous transplantation.

3.3 Assignment of intervention and blinding

Treatment allocation will be performed 24 hour-a-day online by a central randomization web-service. To randomize a patient, investigators have to login, enter the patient identification, and check the inclusion and exclusion criteria. The patient identification must be unique within all randomizations performed by the randomizing investigator's centre. If everything is entered correctly, a summary of the patient data including the randomization result is shown. For double-blind trial the randomization result will be a treatment code. The PI won’t have access to the list of treatment codes used for blinding. Thereby, the treatment code of the patient will be transmited to local pharmacy.

Blinding or masking will be carried out at all stages of packaging and conditioning for shipping following the treatment allocation. Injections used for all groups will be clear and indistinguishable from each other.

During the application of the cells, in the control group, patients will receive the sterile vehicle using similar procedure by unblinded physician (sham procedure without discal injection). The investigator in charge if patient inclusion and follow-up (different from the physician injecting the cells) and the patient will be blinded.

3.4 Patient follow up/Monitoring

Patient follow up will be performed at screening and randomization and at 6 and 12, for 24 months including Clinical parameters (VAS, ODI, SF-36) and MRI at months 6, 12 and 24.

3.5 Withdrawal of the clinical trial

Patients may always and without specification of reasons withdraw their informed consent and, as a corollary, withdraw from the trial related therapy or the entire clinical trial. The investigator also has the right to withdraw patients in agreement with the sponsor or his delegate.

Patients must be withdrawn from the study under the following circumstances:

- The patient withdraws consent,
- Loss of contact to the patient after 3 reminders,
- Development of an intercurrent illness or condition, which would interfere with the patients continued participation,
- Occurrence of a SAE or an exclusion criteria, which would interfere with the patients continued participation,

Discovery that the subject entered the clinical trial in violation of the protocol or occurrence of a significant protocol violation during the clinical trial. The withdrawal will be decided after notice of the principal investigator and methodologist.

Other reason in the opinion of the investigator that cells should not be injected to prevent the patient from harm.

Any situation that in the opinion of the investigator would pose inacceptable risks to the patient if trial participation is continued.

For any discontinuation the investigator should obtain all required details and should document the date and reason of the premature termination in the CRF.

If the reason for withdrawal is an AE, the specific event will be recorded in the eCRF. The investigator will make thorough efforts to document the outcome.

If a patient is withdrawn from the clinical trial before injection of the cells, it will be replaced by another eligible patient.

If a pregnancy occurs, the patient will be withdrawn from the clinical trial and will be closely followed up.

3.6 Lost to follow up

Investigators should make every effort to minimize the number of patients lost to follow-up and to obtain a maximum of information on patients lost to follow-up, particularly in the search for any AEs. Contact details from investigator centre will be given to each patient. The investigator will call the patient before each scheduled visit. If necessary, the investigator could contact General Practitioner (GP) to reach the patient.

3.7 Follow up after discontinuation

If a patient discontinues from the study for an AE, this patient must be followed up until LPLV and until resolution or stabilization of the event.

As far as possible, in case of withdrawal, the investigator will perform all examinations scheduled for the final study visit, which includes recording of AEs. In any case, the patient will be treated in accordance with standard care in the centre.

3.8 Replacement of patients

Drop-outs will not be replaced (the sample size has been increased assuming a drop-out rate of 10%).

If a patient is withdrawn from the clinical trial before injection of the cells, it will be replaced by another eligible patient.

3.9 Follow up after termination

Patients will continue to be followed according to normal care provided by good clinical

practice.

4. Measurement of outcomes

4.1 Primary Outcome Measures:

The clinical response is defined as pain relief of at least 40% and 40 mm decrease on VAS scale between baseline and month 24, or 40% improvement of functional index ODI at month 24 compared to baseline. Chronic LBP is assessed using the VAS pain scale (0 – 100, where 0 represents no pain and 100 represents the worst pain imaginable). ODI scale ranges from 0 to 50 and allows evaluation of disability (0% – 20%: minimal disability; 20% – 40%: moderate disability; 40% – 60%: severe disability; 60% – 80%: crippled; 80% – 100%: bed-bound or exaggerating their symptoms).

4.2 Secondary Outcome Measures

Disability and quality of life evolution include Short Form (SF)-36 scores, global assessment by the patient and the physician. Overall pain intensity in the lumbar spine (1 = none, 2 = mild, 3 = moderate, 4 = severe, 5 = extreme); patient's global assessment of disease activity (1 = very good, 2 = good, 3 = fair, 4 = poor, 5 = very poor); physician's global assessment of disease activity (1 = very good, 2 = good, 3 = fair, 4 = poor, 5 = very poor) will be performed at 0, 3, 6, 12 and 24 months.

Additionally we will assess: de-/increase in rescue painkillers medication. Rescue medication use will be recorded throughout the study duration by a diary file. During the treatment phase of the study, a daily maximum of 3 g

paracetamol/acetaminophen will be permitted. Opioid intake will be possible if paracetamol/acetaminophen is not sufficient. Only tramadol will be authorized.

Pain

The measurement of pain will be determined by self-report of current pain in rest and in motion in the low back pain using a VAS scale. In brief, the VAS consisted of a 10 cm horizontal line anchored with descriptors of pain including "no pain" on one end (left) and "extreme pain" on the other (right). After sitting for 10 minutes, subjects struck a vertical line through the 10 cm VAS representing their CP, and the distance from left end (no pain) to the vertical line was recorded in centimetres. In addition, the patients will fill VAS tests indicating the amount of pain experienced at rest, and in motion.

The drug consumption of painkillers will be assessed throughout the study at each visit. A diary file containing doses, drug name and indication will be given to each patient. The investigator will control this book at each visit. A reduction in dose or frequency of administration of painkillers is an indirect marker of the benefits of MSC therapy.

Employment and work status will be assessed. For this we will assign each of the patients to one of 4 categories designated as “employable” which included those who were unemployed due to pain, employed but on sick leave, laid off, or working. The other categories include retired, disabled, and elderly at least 60 years of age, eligible for social security.

Structural assessment: Evolution of affected disc(s) by quantitative Magnetic Resonance Imaging (MRI) density measurements in T2 and T1spin/echo and T1rho weighted images performed at 0, 6 12 and 24 months used as an indication of disc fluid and GAG content. The "quality" of the patient's lumbar disc will be monitored non invasively using T2weighted MRI sagittal images (Orozco et al., 2011)
and, in T1spin/echo MRI. Lumbar disc grading will be performed in the sagittal T2 weighted images by two physicians independently who were experienced in MRI of the spine. They will review each intervertebral disc from L1–2 to L5-S1 by the modified Pfirrmann criteria. The modified Pfirrmann grading system assesses degenerated intervertebral discs by MRI for the asymmetry in disc structure, distinction of the nucleus and the annulus, signal intensity of intervertebral discs and height of intervertebral discs and assigns grade 1 to 8 for disc degeneration (Table by Griffin et al. Spine 2007).

Table 1. Modified Grading System for Lumbar Disc Degeneration*

Grade	Signal From Nucleus and Inner Fibers of Anulus	Distinction Between Inner and Outer Fibers of Anulus at Posterior Aspect of Disc	Height of Disc
1	Uniformly hyperintense, equal to CSF	Distinct	Normal
2	Hyperintense (>presacral fat and <CSF) ± hypointense intranuclear cleft	Distinct	Normal
3	Hyperintense though <presacral fat	Distinct	Normal
4	Mildly hyperintense (slightly >outer fibers of anulus)	Indistinct	Normal
5	Hypointense (= outer fibers of anulus)	Indistinct	Normal
6	Hypointense	Indistinct	<30% reduction in disc height
7	Hypointense	Indistinct	30%–60% reduction in disc height
8	Hypointense	Indistinct	>60% reduction in disc height

*Grades 1, 2, and 3 are based on the signal intensity of the nucleus and inner fibers of anulus. For Grade 4, the margins between the inner and other fibers of the anulus at the posterior margin of the disc are indistinct. For Grade 5, the disc is uniformly hypointense, although there is no loss of disc space height. For Grades, 6, 7, and 8, there is progressive loss of disc space height. These could be broadly classified as mild, moderate, to severe loss of disc space height. Very occasionally, although obvious disc collapse is present, hyperintense signal from the nucleus and inner fibers of the anulus is preserved. This is referred to by a double entry, *e.g.*, 4/7, with the former reporting the disc signal and the latter the degree of collapse.

Evaluation of cost:

We will compare the medical and non-medical costs between the two groups of patient. Costs will be identified for a one-year time. .

For this purpose, resource use in each arm will be collected in physical units in the eCRF at each clinical centre as follows:

- Acute care medical hospitalisations related to IDD
- Acute care surgical hospitalisations related to IDD
- Rehabilitation hospitalisations related to IDD
- Analgesics
- Work disruption

Safety Outcomes

Prior to enrolment into the trial, subjects will undergo a thorough screening to assess their eligibility for the study, including X-ray and MRI of the lumbar spine, recording of medical history, concomitant medications, vital signs, physical examination, pregnancy test, routine laboratory testing, urinalysis and serological test of infections.

The results from the above mentioned examinations and tests will be used as baseline values and comparators, if and as far as changes of these parameters will occur during the trial.

During each visit to the study centre, participants will undergo a physical examination with recording of vital signs, blood sampling for assessment of routine lab tests, urinalysis, a spine examination and an assessment of pain. Furthermore, if the study patients do not spontaneously report any AE occurrence since their last

visit, they will be interviewed by the investigator filling a study-specific AE checklist for recording of symptoms and complaints.

If an AE occurs at a moment in-between two study centre visits and participants feel that they should consult a physician, they have to call or visit the emergency service of reanimation referring to their participation in the study.

5. Presentation of the Investigational Medicinal Product

5.1 Obtaining, purifying and expanding MSC.

MSC will be obtained following GMP-compliant internal procedure: autologous BM will be seeded w/o any additional manipulation in semi-closed cell culture system (CellSTACK, Macopharma) in Alpha-MEM+10% EDQM certified fetal bovine serum (FBS). MSC will be obtained within 72 hours by plastic adherence; after removal of non-adherent cells, medium will be changed twice a week, w/o neither differentiation nor commitment of cells. Trypzinization will be performed at day 14 and 21 (+/-1 days) using GMP qualified reagents. The final product will undergo to the follow quality controls: Cell count, Immunophenotype, Viability, Endotoxin test (Ph. Eur. 2.6.14), Sterility test (Ph. Eur. 2.6.27), Mycoplasma (Ph. Eur..2.6.7), kariotype. Aliquots are cryopreserved for additional testing if necessary.

5.2 Methodology for obtaining bone marrow autologous

Patient positioning: the patient is in prone position. Bone marrow (BM) is harvested from the posterior superior iliac crest region. Monitored anesthesia (conscious sedation) and local anesthesia will be used. After palpation of the bony landmarks, the procedural site is sterilely prepared and widely draped to ensure an adequate field.

Bone Marrow Aspiration: A Jamshidi needle is used. The skin is then injected down to and including the periosteum with 1% lidocaine without epinephrine. Then, the bone marrow aspiration trochar and needle are percutaneously inserted through the skin and subcutaneous tissues until it reaches the posterior iliac crest. Then, manual pressure is used to position the bone marrow aspiration trochar against the dense cortical bone, centering it over the middle of the posterior crest cortical walls. After the trochar is inserted into the posterior iliac crest but prior to aspiration, 1 mL of heparin (1,000 U/mL) will be preloaded into the syringe. Approximately 20 mL of bone marrow is aspirated, by using two 20-mL syringes. At conclusion a sterile dressing is applied.

The transport container with the raw material, is delivered to the manufacturer. Upon arrival, the container is inspected and the specimen is taken out to the clean-room

for processing. The duration of transport and temperature is recorded. Coherence with accompanying documents is controlled, including donor identifiers.

The sham procedure will consist of the same setting and instruments. The Jamshidi needle will be percutaneously inserted through the skin and subcutaneous tissues and not inserted in the posterior crest .

5.3 Packaging and conditioning for shipping of final product.

The CF-BM-MSC will be prepared in 3 Luer-lock syringes, each containing 2 mL of cell suspension (normal saline solution (0.9% NaCl)+ human albumin 5%) at a cell concentration:

(45±3x10^6 total MSC)

Cell concentration: 7.5 x10^6 cells/mL

That is: 15±1x10^6 cells per syringe in 2 mL

Number of syringes: 3 Total volume: 6 mL

5.4 IMP labelling, storage and handling

The IMP is labeled by the manufacturer according to the applicable regulatory requirements ensuring the traceability and attribution to the individual recipient of the autologous IMP.

The manufacturer ensures a continuous transport of the IMP with the accompaning documents in compliance with the regulatory requirements. A specialized transporter accordingly instructed by the manufacturer delivers the respective IMP to the clinical trial site.

The transport will be done between 4 and 8 °C, with temperature traceability. A suitable mode of transportation ensures delivery of the product to administration site.

5.5 Application of the cells

Material Preparation: Cells tend to aggregate during transport, therefore, prior to application, this effect will be compensated by subjecting the syringe to a gentle oscillating movement allowing the cells to resuspend.

Patient Preparation: It will be verified that the informed consent document, the preoperative tests, and the medical history (that may be of interest during the procedure) are available. An antibiotic prophylaxis will be performed. The prophylaxis is the following: 2g Cefazolin, 1/2 hour before injection. Or in case of allergy: Clindamycin 900 mg (1/2 h before injection). The antibiotic solution is to be delivered intravenously (IV).

Patient positioning: The patient will be placed on a radiolucent surgical table and Wilson frame in a lying prone position. Once the patient is installed, the C-arm mobile radioscopic system will be positioned and tested and the skin prepped.

Intradiscal cellular injection procedure: The injection will be done by fluoroscopy. The investigator enters the images amplifier in the field. The adequate point of entry into the disc will be assessed by fluoroscopy and marked on the skin. The local anaesthesia will be infiltrated. The tip of the needle will advance until it punctures the disc and penetrates the nucleus pulposus. Controls will be carried out in anteroposterior and lateral planes to check the correct position of the needle, prior to the infusion of the cell suspension. After removing the needle mandrel, the syringe will be screwed to the needle and the cellular suspension (2ml containing $15\pm1x10^6$ MSC) will be slowly injected, stopping temporarily if the pain is exacerbated. A small Mepore dressing will be put into place.

5.6 Application of sham procedure

In the other group, the sham procedure will be performed in the same conditions in order to keep blind the patient. The person in charge of injection, who’s the only unblinded investigator will mimic intradiscal injection by using imaging with only paravertebral muscle puncture. No disc injection, no placebo injection will be perfomed in the control patients.

The annulus will not be perforated. A puncture with a needle into deep muscle (Musculus erector spinae) will be performed, however no lidocaine or other fluids will be delivered in the vicinity of the disk, as to avoid any positive or negative bias. As the patient will be lying on the table and covered by the drape, he/she will not be aware that the C-arm is not in action during the procedure.

5.7 Post-treatment care

The patient is discharged after an observation period of 2 hours. Walk for moderate periods is authorized. Labor activity is suspended for one week. The pain medication is tailored to the needs of each patient and the use of NSAIDs should be avoided. The longterm monitoring will consist of out-patient visits and MRI.

The patients are followed for 2 years on clinical examination, spine MRI. For 2 years they will be followed annually on clinical basis to assess any unexpected long term side effect.

In case the patient have not responded after end of study (3 years) or relapse, he may be eligible for cell therapy when the product will be available on routine basis, or benefit for treatment recommended by EULAR (physiotherapy, steroid or surgery)

5.8 Possible complications of this therapy.

The complications are the same as in the discography, and rarely presented. The most common complaint is the exacerbation of pain for 1-2 weeks, which is usually solved by analgesia and muscle relaxants for a short period. The most serious complication is discitis, occurring in less than 0.1 % (Osti et al., 1990). Other complications described, although much less frequently, are transient headache, nausea, meningitis, epidural abscess, arachnoiditis, intrathecal hematoma, retroperitoneal hematoma, cauda equina syndrome and acute disc herniation. Cases of urticaria are not likely in this therapy, as they are attributable to the radiologic contrast, which is not used here. Initial fears about the possible long term side effects of discography regarding the disc viability have not been confirmed. A 20-year of clinical follow-up did not find radiographic evidence of progressive disc degeneration after the discographies (Flanagan & Chung , 1986).

There is no risk of rejection using autologous cells.

6. Study procedures and visits

Each person mus be fully informed prior to being enrolled to the clinical trial. Eligible subjects have to be informed in person by the investigator and in writing by the patient information. Only after clarification of all questions this person is asked to sign two copies of the consent form and date by hand. Then a copy of patient information and informed consent of the eligible person shal be issued; the second copy of the consent form is kept in the ISF.

6.1 Schedule of assessment

CHRONOGRAM	Pre-Screening	Screening	Inclusion/ randomization	BM withdrowel	Baseline Treatment	Tracking (M = months)				
PROGRAMMING OF VISITS:	Day -60/-46	Day -45/-41	Day -45/-31	Day -45/-31	V0	V1	V2	V3	V4	V5
					Day 0	M1 (+/- 7)	M3 (+/- 7)	M6 (+/- 14)	M12 (+/- 30)	M24 (+/- 30)
Instructions for wash-out48 hours NSAIDs[a] washout		X			X	X	X	X	X	X
Prohibition of NSAIDs[a]					X	X	X	X	X	X
Instructions for wash-out 24 hours painkillers[b] washout		X			X	X	X	X	X	X
Delivery of the Information sheet	X									
Signature of Informed Consent form		X								
General medical History and Demography		X								
Lumbar related medical history		X								
Vital signs[c]		X		X	X	X		X	X	X
Physical Exploration		X		X	X	X		X	X	X
RX of Lumbar Spine Simple and Dynamic		X								
ECG 12-Lead		X								
Blood sampling for routine laboratory testing and urine analysis		X		X	X	X	X	X	X	X
Urine pregnancy test		X								
Preceptive Viral Serologies		X								
Checking Inclusion and exclusion criteria		X								
Randomization			X							
Pain lumbar spine (VAS), ODI, SF-36	X	X		X	X	X	X	X	X	X
Lateral MRI, T2, quantitative	X[d]				X			X	X	X
Adverse Events					X	X	X	X	X	X
Concomitant medication		X		X	X	X	X	X	X	X

a: NSAIDs washout of at least 2 days before screening, inclusion and baseline. NSAIDs will not be authorized during the first 6-month after baseline . However, if needed after the first 6 months, NSAIDs could be used

b: Patients will be required to refrain from taking paracetamol or opioids within 24 hours of each clinical visit for efficacy evaluations

c: vital signs: Temperature, heart rate, arterial blood pressure, height, weight

d: MRI was also used to assess the Pfirrmann and Griffith's score

6.2 Visits during the course of the trial

6.2.1 Pre-Screening visit

- Delivery of the Information sheet.
  - Lateral MRI, T2, quantitative.
  - Pain assessment of the lumbar spine by visual pain scale (VAS, Oswestry Disability Index ODI in order to measure a patient's permanent functional disability, Quality of life assessment using SF-36.

6.2.2 Screening visit

- Signature of Informed Consent Form.
- Checking inclusion and exclusion criteria.
- Demografics and general medical history.
- Lumbar related medical history (includes past history of osteoarthris, osteoporosis, lumbar pain, injury, trauma, fractures, chirurgical procedure or other conditions.
- Medication history therapy and unresponsive (including physical therapy) for at least 6 months.
- Vital signs after sitting for at least 5 minutes, (temperature, blood pressure, and heart rate, height, weight).
- Musculoskeletal physical exploration.
- Lumbar spine examination (X ray).
- Pain assessment of the lumbar spine by visual pain scale (VAS, Oswestry Disability Index ODI in order to measure a patient's permanent functional disability, Quality of life assessment using SF-36.
  - Instruction for wash out (NSAIDS washout of at least 2 days before the screening Visit, no painkillers 24h before the screening visit) will be given to the patient after written informed consent.
- If clinical and radiological criteria fulfilled, perform ECG (12-lead)
- Blood sampling for routine laboratory testing

  Haematology: haematocrit, haemoglobin, RBC, WBC; PC

  Biochemistry: Electrolytes, Creatinine, BUN, ALT, AST, AP, Bilirubin,

  Coagulation (INR, PTT)

  CRP
- Urinalysis (dipstick): Glucose, Ketones, blood, protein, nitrite, leucocytes, , bilirubin in urine
  - Urine pregnancy test in woman of childbearing

potential

- Blood sampling for preceptive viral serologies

  HBs antigens

  Anti-HBc antibodies

  Anti-HCV antibodies

  Anti-HIV-1 and anti-HIV-2 antibodies, Anti HV1 gp41

  Anti-HTLV-1, HTLV-2 antibodies

  Wasserman testing

Blood testing for Syphilis serology

- Concomitant medication

6.2.3 Inclusion visit and randomization

- Confirm inclusion and exclusion criteria
- Randomization

Randomization procedure: the randomization is performed 24 hour-a-day online by the central randomization web-service customized by the sistem. To randomize a patient, investigators have to login, enter the patient identification, additional checks and factor values. The person in charge of cell injections will be strictly forbidden from discussing treatment allocation with patients and clinical observers.

6.2.4 BM-MSC Withdrawall

- Prior to Bone Marrow cells extraction:

- Physical exploration
- Vital signs (Temp, BP, HR)
- Blood sampling for routine laboratory testing

Haematology: haematocrit, haemoglobin, RBC, WBC; PC

Biochemistry: Electrolytes, Creatinine, BUN, ALT, AST, AP, Bilirubin, CRP

- Urinalysis (dipstick): Glucose, Ketones, blood, protein, nitrite, leucocytes, bilirubin in urine

- Pain assessment of the lumbar spine by visual pain scale (VAS, Oswestry Disability Index in order to measure a patient's permanent functional disability, Quality of life assessment using SF-36
- An antibiotic prophylaxis will be performed. The prophylaxis is the following: 2g Cefazolin, 1/2 hour before injection. Or in case of allergy: Clindamycin 900 mg (1/2 h before injection). The antibiotic solution is to be delivered intravenously (IV).
- Concomitant medication (analgesic medication with diary file)

Bone Morrow Cell extraction procedure

The surgical procedure is described in section 5.3

After Bone Morrow Cell extraction procedure

After completion of the BM extraction procedure subjects are monitored for vital signs for 2 hours and they will have an authorization to leave the investigation centre after a last check by the investigator.

Prior to discharge a physical examination of the subject and AE recording is performed by the investigator.

A second visit 2’ equivalent to the visit 2 will be done if a problem occurs as cells contamination shipping problem, not enough cells after the first bone-aspiration.

6.2.5 Day 0 - Visit 0: Visit baseline and treatment

- Physical exploration
- Vital signs (Temp, BP, HR)
- Lateral MRI, T2, quantitative

- Medication history therapy
- Blood sampling for routine laboratory testing
- Urine analysis

- Pain assessment of the lumbar spine by visual pain scale (VAS)
- Oswestry Disability Index in order to measure a patient's permanent functional disability, Quality of life assessment using SF-36
- Instruction for wash out (NSAIDS washout of at least 2 days before this screening Visit, no painkillers 24h before the visit)
- Prohibition of NSAIDsa
- Adverse event assessment
- Concomitant medication
- Intervention : injection of autologous cells or sham procedure (under fluoroscopy guide and monitor cell implantation). The affected disks (max 3) are treated simultaneously once only, at the V0 visit and no subsequent doses are expected.

6.2.6 Visit 1: Month 1

- Vital signs
- Physical exploration
- Blood sampling for routine laboratory testing
- Urinalysis
- Pain assessment of the lumbar spine by visual pain scale (VAS)
- Oswestry Disability Index in order to measure a patient's permanent functional disability, Quality of life assessment using SF-36
- Adverse event assessment
- Instruction for wash out (NSAIDS washout of at least 2 days before this Visit, no painkillers 24h before the visit)
- Prohibition of NSAIDsa
- Concomitant medication

6.2.7 Visit 2: Month 3

- Blood sampling for routine laboratory testing
- Urinalysis
- Pain assessment of the lumbar spine by visual pain scale (VAS)

- Oswestry Disability Index in order to measure a patient's permanent functional disability, Quality of life assessment using SF-36
- Adverse event assessment
- Instruction for wash out (NSAIDS washout of at least 2 days before this Visit, no painkillers 24h before the visit)
- Prohibition of NSAIDsa
- Concomitant medication

6.2.8 Visit 3: Month 6

- Vital signs
- Physical exploration
- Blood sampling for routine laboratory testing
- Urinalysis
- Lateral MRI, T2, quantitative
- Pain assessment of the lumbar spine by visual pain scale (VAS)
- Oswestry Disability Index in order to measure a patient's permanent functional disability, Quality of life assessment using SF-36
- Adverse event assessment
- Concomitant medication
- Instruction for wash out (NSAIDS washout of at least 2 days before this Visit, no painkillers 24h before the visit)
- Prohibition of NSAIDsa

6.2.9 Visit 4: Month 12

- Vital signs
- Physical exploration
- Blood sampling for routine laboratory testing
- Urinalysis
- Lateral MRI, T2, quantitative
- Pain assessment of the lumbar spine by visual pain scale (VAS)
- Oswestry Disability Index in order to measure a patient's permanent functional disability, Quality of life assessment using SF-36
- Adverse event assessment

- Concomitant medication
- Instruction for wash out (NSAIDS washout of at least 2 days before this Visit, no painkillers 24h before the visit)
- Prohibition of NSAIDsa

6.2.10 Visit 5: Month 24

- Vital signs
- Physical exploration
- Blood sampling for routine laboratory testing
- Urinalysis
- Lateral MRI, T2, quantitative
- Pain assessment of the lumbar spine by visual pain scale (VAS)
- Oswestry Disability Index in order to measure a patient's permanent functional disability, Quality of life assessment using SF-36
- Adverse event assessment
- Concomitant medication
- Instruction for wash out (NSAIDS washout of at least 2 days before this Visit, no painkillers 24h before the visit)
- Prohibition of NSAIDsa

6.3 Unscheduled visits

If subject gets in contact with the site between regular visits due to acute disorders, an unscheduled visit will promptly be performed at the site. During this additional visit a physical examination, a systematic interview for recording AEs should be performed. In case of suspected local infection (nocturn lumbar pain, temperature above 38°C), disc puncture will be performed and an empirical antibiotic treatment will be initiated immediately and further tuned according to the antibiogram

6.4 Concomitant therapy and Rescue therapy

Rehabilitation: Lifestyle modification, particularly exercise and weight reduction, is a core component of the management of low back pain.

Rescue medication use will be recorded for the study duration. Within 24 hours following MSC injection, paracetamol/acetaminophen will be advised for each patient if needed.

Patients will be required to refrain from taking paracetamol or opioids within 24 hours of each clinical visit for efficacy evaluations. If the patient required, supplementary analgesic medication will be provided Tramadol will be used as rescue medication.

NSAIDs will not be authorized during the first 6-month of the study. However, if needed after the first 6 months, NSAIDs could be used.

A diary file will be provided for each patient in order to collect information on medications use, mobility, activities and rehabilitation sessions.

6.5 Contraceptive methods

Adult subjects of childbearing potential (ACBP) must:

- Understand the potential teratogenic risk to the unborn child and the need for effective contraception.
- Be capable of complying with effective contraceptive measures.
- Be informed and understand the potential consequences of pregnancy and the need to notify her study doctor immediately if there is a risk of pregnancy.
- Understand the need to commence the study treatment as soon as study drug is dispensed following a negative pregnancy test.
- Understand the need and accept to undergo pregnancy testing based on the frequency outlined in this protocol.
- ACBP enrolled in this protocol must agree to use two reliable forms of contraception simultaneously or to practice complete abstinence from heterosexual contact during the following time periods related to this study: 1) while participating in the study; 2) in case of dose interruptions; and 3) for at least 24 months after the last dose of study drug.
- The two methods of reliable contraception must include one highly effective method and one additional effective (barrier) method. ACBP must be referred to a qualified provider of contraceptive methods if needed. The following are examples of highly effective and additional effective methods of contraception.

Highly effective methods:

- *Hormonal (birth control pills, injections, implants)
- Tubal ligation
- Partner's vasectomy (if medical assessment ascertain the surgical success)
- Intrauterine Device (IUD)
- Intrauterine hormone-releasing system (IUS)

- Bilateral tubal occlusion
- Sexual abstinence (if defined as refraining from heterosexual intercourse during the entire period of risk associated with the study treatments. The reliability of sexual abstinence needs to be evaluated in relation to the duration of the clinical trial and the preferred and usual lifestyle of the subject.)

-**Additional effective methods:**

- Male condom
- Diaphragm (not recommended in patients with low platelets count)
- Cervical cap.

Males with ACBP Partners:

- Condom
- Contracpection durin treatment and until the end of relevant systemic exposure in ACBP.

Definition:

Women of childbearing potential a woman is considered of childbearing potential (WOCBP), i.e. fertile, following menarche and until becoming post-menopausal unless permanently sterile. Permanent sterilisation methods include hysterectomy, bilateral salpingectomy and bilateral oophorectomy.

A postmenopausal state is defined as no menses for 12 months without an alternative medical cause. A high follicle stimulating hormone (FSH) level in the postmenopausal range may be used to confirm a post-menopausal state in women not using hormonal contraception or hormonal replacement therapy. However in the absence of 12 months of amenorrhea, a single FSH measurement is insufficient.

A man is considered fertile after puberty unless permanently sterile by bilateral orchidectomy.

7. Statistic analysis planning and power calculation

Sample size was calculated to have 80% power and 2 balanced groups and according to the literature data (Maher et al., 2017; PopulationReferenceBureau; 2016), a responder rate is expected at 12 months of the order of 30% in controls and 68% for MSC is a delta of 38% between the two groups. To highlight this

difference while justifying a power of 80% and an alpha risk of 5% and within 1:1 ratio, taking into account 20% of inclusion failure, it is necessary to include 26 individuals per group a total of 52 subjects.

7.1 Statistic analysis

Evaluation of the treatment effect will be obtained with analysis of variance (ANOVA) and Student T test.

7.2 Sensitivity analysis

Sensitivity analysis will be performed in the full data set population (including all subjects who are randomized, receive the injection, and have a valid primary efficacy baseline measurement and at least one valid post-baseline primary efficacy measurement). We will also test a worst-case maximum bias scenario, in which we assume that all patients with missing data are not responders in the intervention group and responders in placebo group.

Multivariates regression will be used to test the impact of covariates not comparable at baseline, and to test the impact of a rehabilitation program attended during the follow-up.

7.3 Safety analysis

Adverse events that occur during this study will be presented by system organ class (SOC), high level term (HLT), and preferred term (PT) in a frequency table giving the number of events, the number of subjects, and the percentage of subjects who experience the event by treatment group.

Subjects with multiple AEs will be counted only once within each PT, HLT, and SOC.
Coding of the AEs will be performed with the Medical Dictionary for Regulatory Activities.

7.4 Handling of protocol deviations

The clinical project manager will prepare the Specification of Protocol Deviations in consultation with the Clinical Trial Biostatistician and Study Physician. Search criteria for potential protocol deviations will be detailed in the Specification of Protocol Deviations. During the blind period, the protocol deviations will be classified according to their impact on the primary objective.

All important protocol deviations, including missing relevant data, will be listed by subject.

7.5 Handling of dropouts or missing data

Rules for imputation of missing data will be detailed in the SAP. When analyzing efficacy data, if the missing data is type MAR (Missing At Random) or MCAR (Missing Completely At Random), a multiple imputation will be implemented.

8. Assessment of Safety and Tolerability

Safety and tolerability will be evaluated by recording adverse events (AEs) and serious AEs (SAEs) throughout the study, on all patients enrolled in the study. Number of participants with adverse events, type of adverse event and their repartition between treatment arms will be used as a measure of safety and tolerability.

8.1 Safety parameters

The following parameters will be assessed and analysed for study product safety:

- clinical review and questionnaires for pain,
- disability and quality of life at 0, 6 12 and 24 months
- physical examinations performed at baseline , M3, M6, M12 and M24 after injection of the cells;
- laboratory tests (haematology, blood chemistry) and vital signs assessed at baseline, M1, M3, M6, M12 and M24. Elevations in ALT and/or AST >3 × ULN will also be reported since this level was considered to be most relevant to health authorities and healthcare providers.

8.2 Definition

Adverse Event :

An adverse event is any untoward medical occurrence in a patient or clinical investigation subject administered a pharmaceutical product and which does not necessarily have to have a causal relationship with this treatment. An adverse event (AE) can therefore be any unfavourable and unintended sign (including an abnormal laboratory finding, for example), symptom, or disease temporally associated with the use of a medicinal product, whether or not considered related to the medicinal product.

An adverse event, whether or not considered to be the causally related to the investigational medicinal product, may be:

The deterioration of a pre-existing chronic disease or aggravation of a symptom or disease that was present on enrolment of the patient in the study

A symptom or disease discovered after the start of the study even if it was probably present prior to the patient's enrolment in the study.

Abnormal laboratory findings will be considered as adverse event when they are considered /judged as clinically significant by the investigator.

Clinically significant event/value should be defined as: symptomatic, requiring corrective treatment, leading to discontinuation, dose delay, dose reduction, dose interruption and/or fulfilling seriousness criteria.

Adverse drug reaction (ADR)

All noxious and unintended responses to a medicinal product related to any dose should be considered adverse drug reactions.

An adverse reaction is an AE that is at least possibly related to the administration of the study medication or procedure. When assessing the relationship (“related” or “unrelated”) between an AE and the study medication or procedure, the following points should be taken into consideration:

- close timely relationship between administration of study medication or procedure and occurrence of AE
- information about the side effect profile of the study medication or procedure from non-clinical and clinical studies
- mechanism of action of study medication or procedure potentially explaining the occurrence of the AE
- other factors with causal potential
- impact of concurrent therapeutic or diagnostic measures
- personal physical or psychological stress factors on the participant’s side

Serious adverse event (SAE)

A SAE is an AE with at least one of the following characteristics:

- results in death
- is life-threatening
- requires inpatient hospitalization or prolongation of existing hospitalization
- results in persistent or significant disability or incapacity
- is a congenital anomaly or birth defect
- is an important medical event

NOTE: The following hospitalizations are not considered SAEs:

- a visit to the emergency room or other hospital department lasting less than 24 hours that does not result in admission (unless considered an “important medical event” or a life-threatening event)

- elective surgery planned before signing consent
- admissions as per protocol for a planned medical/surgical procedure
- routine health assessment requiring admission for baseline/trending of health status (eg, routine colonoscopy)
- medical/surgical admission for purpose other than remedying ill health state that was planned before study entry. Appropriate documentation is required in these cases.
- admission encountered for another life circumstance that carries no bearing on health status and requires no medical/surgical intervention (eg, lack of housing, economic inadequacy, caregiver respite, family circumstances, administrative).

Serious adverse reaction (SAR)

A SAR is an AE which is at least possibly related to the study medication or procedure and fulfils at least one of the criteria of seriousness listed above.

A suspected expected serious adverse reaction (SESAR) during a clinical trial is an AE which is at least possibly related to the study medication or procedure, fulfills at least one of the criteria of seriousness listed above and is listed as potential side effect of the study medication or procedure in the Summary of Product Characteristics or the Investigators' Brochure.

A suspected unexpected serious adverse reaction (SUSAR) during a clinical trial is an AE which is at least possibly related to the study medication, fulfils at least one of the criteria of seriousness listed above and is not listed as potential side effect of the study medication or procedure in the Summary of Product Characteristics.

New events

A new fact is defined as any new safety data, which could lead to a reassessment of the risk/benefit balance of the study or of the investigational product or that could be sufficient to consider changes in the drug administration or in the pursuit of the study

8.3 Expected adverse reactions

Reference safety document: The current Investigator brochure will be considered as the reference safety document.

Expected Adverse Reaction related to injection of autologous bone marrow mesenchymal cell (undetermined frequency, not severe except for immunological reaction)

- transient lumbar pain
- elevation of the body temperature (>37,5°C)

- shivering
- pruritus

Expected Adverse Reaction related to local intra-discal injection

- headache and nausea (undetermined frequency, not severe) The following adverse reactions are severe but rare
- local infection (discitis),
- reherniation in operated segments,
- exacerbation of pain, back pain, ischialgia, sciatica, pain in extremity,
- facet joint syndrome,
- meningitis,
- epidural abscess,
- arachnoiditis,
- intrathecal hematoma,
- retroperitoneal hematoma,
- cauda equina syndrome,
- acute disc herniation,
- paralysis,
- cerebrospinal fluid leak.

Expected Adverse Reaction related to the concomitant medication

Cf. the current Summary of Product Characteristics (SmPC) to the product.

Expected Adverse Reaction related to MRI

Panic attack

Expected Adverse Reaction related to local anesthesia

- Related to local anesthesic or antiseptic :

Cf. the current Summary of Product Characteristics (SmPC) to the product (1% xylocain for local anesthesic, and chlorhexidine for antiseptic)

- Related to the procedure/technique of local anesthesia:

Anesthesia failures and insufficiencies

Hematomas and arterial dissections

Infections

Neurological lesions (traumatic nerve location or intraneural injection or injected solution toxic)

Error on injected product (Ex: antiseptic, physiological saline)

8.4 Unblinding procedure

This trial is a double blind clinical study for both patient and investigator in charge of the patient evaluations. The knowledge by investigator of the treatment administrated in the study may be necessary but should be exceptional and justified in case of SAEs or specific situations by the adaptation of patient care and medical care depending on the treatment received

SAEs and / or situations that could justify unblinding request on medication administrated as part of the protocol are defined below:

a. Infections requiring hospitalization and administration of IV antibiotics:

b. Unexplained or possibly toxic death.

c. Request by health professional (emergency room, intensive care ...) not involved in the study, the patient himself or his entourage in an immediate emergency.

Concerned patient by unblinding procedure will not be withdrawn of the study

8.5 Investigator responsibilities

AEs

Complete and appropriate data on all AEs (only clinically significant event or value) experienced during the clinical trial should be recorded on the AE form of the CRF on an ongoing basis for the duration of the study. Each AE report shall include a description of the event, an assessment of its seriousness according to the criteria listed above, its duration, intensity, relationship to the study medication, other causality factors (if any), any concomitant medication dispensed, actions taken with the study drug or other therapeutic interventions and outcome at the end of the observation period.

The adverse events will be encoded and graded according to CTCAE Criteria Grade 1-5
(Common Terminology Criteria for Adverse Events). A copy of the CTCAE Version 5.0 can be downloaded from the CTEP homepage (http://ctep.info.nih.gov). If there is no CTCAE grading possible, the intensity of the adverse event has to be classified in categories:

- Mild (Grade 1) – transient or minimal symptoms; no change in activity or need of medication
- Moderate (Grade 2) – symptomatic; moderate change in activity; no reduction in social activities

- Severe (Grade 3) – incapacitating; bed rest required; loss of work; reduction in social activities
- Life threatening (Grade 4) – significant clinical intervention or hospitalization required
- Fatal (Grade 5)

For each AE, a separate AE form will be filled in.

Follow up of AEs: AEs will be followed until their resolution or stabilisation. However, the observation period will be cut off after the last patient has finished his final visit (LPLV). During monitoring visits, the study monitor will assess accuracy and completeness of all AE forms generated since the last visit by 100% source data verification. Cases of AEs reported earlier for which the outcome is still pending will be checked for new data and developments. Furthermore, queries on AEs reported earlier will be solved with the Investigator. AEs will be reported in the final study report.

The sponsor must be informed immediately of the occurrence of any AE grade 3-4-5 by the associated centre.

SAEs

The Investigator must also record all SAEs in the CRF. For each SAE an additional paper based SAE-Form has to be filled in.

The investigator shall report all SAE immediately involving study participants, including suspected predefined gastro-intestinal, cerebro-vascular and/or hepatic events for assessment and adjudication in a blinded manner by the vigilance department of the sponsor, except for those that the protocol identifies as not requiring immediate reporting such as:

- Specific hospitalisation (Definition of SAEs)
- Serious adverse events occurring after given informed consents, but treatment administration (Visit 1).

These identified SAE will be documented on the CRF/ source data as AE.

The Pharmacovigilance Clinical Trial Department of the sponsor must be informed immediately (i.e. within 24 hours) of the occurrence of any SAE by fax or e-mail (contact details see below).

The Pharmacovigilance Department will confirm receipt of the initial SAE report by return fax or e-mail and provide a SAE case identification number. As on the CRFs, data should be transmitted in pseudonymised form, yet allow identification of the subject by the Investigator.

Causality assessment: The Investigator is required to include any investigations as may be indicated to elucidate the nature of or the causality of the SAE. This may include additional lab tests, histo-pathological examinations, and consultations with other healthcare professional or if the patient dies, any post mortem findings.

Follow up: After the initial report, the Investigator is required to follow each patient and to provide further information on the patient's condition to the sponsor. The Investigator will ensure that the follow-up includes any further investigations as may be indicated to elucidate the nature of the causality of the SAE. This may include additional lab tests, histo-pathological examinations, and consultations with other healthcare professional or if the patient dies, any post mortem findings.

Pharmacovigilance: Dr. Andrea Di Mattia

FAX number: +39 06 225411157

E-mail: a.dimattia@unicampus.it

8.6 Sponsor responsibilities

Serious Adverse Event and new event

The relation between the event and the study drugs should be evaluated by the sponsor. The sponsor will evaluate whether the events are expected or unexpected.

The sponsor will report any SUSAR and new events according to the legislation and good clinical practice. Any Serious Adverse Reaction related to injection of allogeneic bone marrow mesenchymal cell, to graft rejection of allogeneic bone marrow mesenchymal cell, to local intra-discal injection and those related to bone marrow donation will be declared to the corresponding national competent authority in the same way that SUSARs Regulatory declaration is made within a maximum of:

- without delay for serious adverse unexpected fatal or life-threatening. In this case, additional relevant information should be sought and passed within a further period of 8 days.
- 15 calendar days for all other serious unintended effects. The same additional relevant information should be sought and transmitted in a further period of 8 days.

Assessment of Adverse Events

The assessment of the relationship of an AE to the administration of Drug Product is a clinical decision based on all available information at the time of, and after the occurrence of the event. The factors to be considered when evaluating the relationship of an AE to the Drug Product include:

- Temporal sequence from drug administration: the event must occur after the Drug Product is given. The length of time from exposure to medication to event should be evaluated in the clinical context of the event.
- Recovery on discontinuation (dechallenge), recurrence on reintroduction (rechallenge).

- Underlying, concomitant, intercurrent diseases: each report should be evaluated in the context of the natural history and course of the disease being treated and any other diseases the patient may have had prior to, or developed during the course of the study.
- Concomitant medication or treatment: the other drugs the patient is taking or the treatment the patient is receiving at the time of the event should be examined to determine whether any of them may be recognized to cause the event in question.
- Known response pattern for this class of drug.
- Exposure to physical and/or mental stress: the exposure to stress may induce adverse changes in the patient and may provide a logical explanation for the event.
- The pharmacology and pharmacokinetics of the Drug Product: absorption, distribution, metabolism and excretion of the Drug Product or other medications the patient is receiving, coupled with the pharmacodynamic responses, should be considered when evaluating an event. From a statistical and regulatory point of view, an AE will be categorized as "reasonable" or "not reasonable" related to Drug Product.

Expedited reporting

Other safety issues also qualify for expedited reporting where they might materially alter the current benefit-risk assessment of the investigational medicinal product or that would be sufficient to consider changes in the investigational medicinal products administration or in the overall conduct of the trial, for instance:

a) an increase in the rate of occurrence or a qualitative change of an expected serious adverse reaction (SESAR), which is judged to be clinically important,
b) post-study SUSARs that occur after the patient has completed a clinical trial and are reported by the investigator to the sponsor, up to 1 year follow up
c) new events related to the conduct of the trial or the development of the investigational medicinal products and likely to affect the safety of the subjects, such as:
d) a SAE which could be associated with the trial procedures and which could modify the conduct of the trial,
e) a major safety finding from a newly completed animal study (such as carcinogenicity)

Development Safety Update Report (DSUR)

In addition to the expedited reporting, the sponsor shall submit, once a year (on the anniversary of the first approval date of the clinical trial by regulatory authorities), throughout the clinical trial or on request, a safety report to the competent authorities

and the Ethics Committee of the concerned Member States taking into account all new available safety information received during the reporting period.

The aim of the DSUR is to describe concisely all new safety information relevant for one or several clinical trial(s) and to assess the safety conditions of subjects included in the concerned trial(s).

It should be the same for the competent authorities concerned and the Ethics Committee concerned.

The Sponsor is responsible for the ongoing safety evaluation of the Investigational Product. The Sponsor should promptly notify all concerned investigators and the regulatory authorities of findings that could affect adversely the safety of subjects, impact the conduct of the trial, or alter the CA approval/ EC's favourable opinion to continue the trial.

*8.*7 Data and Safety Monitoring Board

No independent data monitoring committee has been appointed for this study.

No formal interim statistical analysis will be performed.

9 Data handling and record keeping

It is the responsibility of the Sponsor to ensure that the clinical trial is conducted according to all stipulations of the protocol and in accordance with ICH-GCP, the Declaration of Helsinki and local regulatory requirements.

The Sponsor or his designee must ensure that data are recorded in the eCRF correctly and completely by authorized personnel. The investigator has to confirm the integrity of the data transferred to the eCRF by signature.

The investigator is responsible for the completion and maintenance of the confidential patient identification code which provides the unique link between named patient source records and pseudonymized eCRF data. The investigator must arrange for the retention of this patient identification log in the ISF..

The principal investigator of the site must provide a staff signature list to determine the responsibilities of each person of the trial personnel. The staff signature list has to be kept in the ISF and TMF.

9.1 Investigator site file (ISF)

The investigator is responsible for maintaining all records which enable the conduct of the clinical trial at the site to be fully documented, in compliance with ICH GCP filing standard. Timeliness and completeness of the documentation is regularly checked by the clinical monitor.

9.2 Trial Master file (TMF)

The sponor correspondent for the clinical trial management (regular submissions/visits) will maintain the TMF with relevant document of the clinical trial according to ICH/GCP filling standard.

9.3 Obligation to archive (Principal investigators and sponsor)

All completed study related documents (e.g. eCRF, Informed consent forms, drug accountability logs, staff signature lists, Subject identification log, study initiation visit and monitoring visits reports) must be archived by each investigator for its centre and by the sponsor for 30 years.

9.4 Adherence to the clinical trial protocol

Protocol violations are any deviations from the procedures outlined in this document, for example, missed evaluations, incorrect timing of evaluations, non-compliance with GCP and intake of prohibited medications. It is the investigator's responsibility to make all reasonable efforts to avoid protocol violations in order to avoid possible exclusion of the patient from the study and/or analyses.

An effort should be made to ask the sponsor for permission to deviate from the protocol if really necessary. Only if the safety of a patient is in immediate danger, the investigator may deviate from the protocol on his own responsibility.

All protocol violations will be reported immediately to the Sponsor or his delegate and any action required, for example, discontinuation of the patient will be discussed.

Any deviations from the protocol that has not been approved by the Sponsor or his delegate, the Competent Authority and the concerned EC could result in a discontinuation from the study of the site involved.

10 Data collection and data management

Data collection will be performed with electronic Case Report Forms (eCRF). The data management will follow a Remote Data Entry approach. The eCRF will be implemented in a modern Clinical Data Management System (CDMS) with Electronical Data Capture functionality (EDC).

-. During the trial all findings will be documented on eCRFs or other study specific paper forms by the responsible investigator or designated representatives. The investigator will maintain a list of these representatives. Paper forms will be signed by either of them. The data has to be complete, clear, accurate, legible, and plausible. Missing examinations or dates have to be marked along with a justification/explanation. Corrections on study paper forms are to be made according to GCP guidelines, i.e. the version that has to be corrected will be crossed in a way that it is still readable, the corrected version will be written above or beside the final

version and the correction (or any remark) will be marked with data, initials, and a justification by the investigator or an authorized person. The query management is performed electronically under the supervision of the monitor. Data corrections in the eCRF, if necessary, have to be performed by the Investigator or designated representatives. Only these persons are allowed to the system and their identity during use will be registered. Data on patients collected on eCRFs in the course of the trial will be documented in a pseudonymous fashion. For monitoring and auditing purposes, and to the greatest extent possible, all information must be traceable back to the source documents, which are generally maintained in the patient’s file. The source documents should cover demographic and medical information, including laboratory data, medication, physical examination, etc.

11 Quality control and quality assurance

11.1 Control of data consistency

Computerized and manual consistency checks will be performed on newly entered forms; queries will be issued in case of inconsistencies. Consistent forms will be validated by the Data Manager to be entered on the master database. Inconsistent forms will be kept "pending" until resolution of the inconsistencies.

11.2 On-site quality control

In order to censure that the study is conducted according to Good Clinical Practice (GCP), the Sponsor will send to clinical center an Investigator’s File, will organize Training Meetings in which principal investigators as well as collaborative investigators will be involved. In these meetings the following issues will be addressed:

1. Regulatory procedures.
2. Compulsory documents to be sent to the Sponsor in order to be authorized to enrol patients.
3. Study documents archive system.
4. Patient information sheet and informed consent: how to approach the patient and where to archive the document.
5. Patient selection criteria and registration procedure.
6. CRFs, queries management.
7. SAEs/SUSARs
8. Main source documents to be sent to the Sponsor.

During the first Training Meeting, different operative procedures will be distributed and explained, procedures concerning patient selection criteria and registration procedures, CRFs and SAEs/SUSARs. All these will be in the Investigator's File as well.

During the general meetings, a report concerning the conduction of the study will be distributed.

In this report, up to date data can be found concerning not only accrual but also SAEs/SUSARs, list of participating centers and particular situations that may have arisen during the conduction of the trial. This report constitutes an important working tool for the Investigator and is also an up to date report to be periodically presented to the Ethics Committee.

Furthermore, the statistical design and the precise accrual will be a method to select data that need to be verified.

12 Monitoring, audits and inspections

12.1 Monitoring

Monitoring will be performed during the whole study at investigating sites and pharmacies according to the sponsor specific SOP (monitoring plan). The monitoring will include clinical parameters (VAS, Oswestry, SF-36 questionnaires), MRI, AE.

Routine monitoring visits will be made by the monitors designated by the Sponsor to check compliance with the protocol, the completeness, accuracy and consistency of the data, and adherence to GCP.

The principal investigator must ensure that eCRFs are completed in a timely manner and must allow periodical access to eCRFs, patient records, drug logs and all other studyrelated documents and materials. The frequency of monitoring visits will be determined by factors such as study design and the site enrolment requirements, according to monitoring plan.

The investigator will agree to provide the monitor direct access to the subjects' source data, which may exist in the form of hospital records, patient files and notes, and laboratory assessment reports and results.

12.2 Audit and Inspection

The purpose of an audit is to confirm that the study is conducted as per protocol, ICH-GCP and applicable regulatory requirements, that the well-being and the rights of the subjects enrolled have been protected, and that the data relevant for the evaluation of the investigational Medical product have been recorded, processed, and reported in compliance with the planned arrangements. The investigators will permit a direct access to all study documents, drug accountability records, medical records and source data.

13 Informed Consent, Ethical Review, and Regulatory Considerations

13.1 Informed consent

Informed consent must be obtained from each subject, before the performance of any study-related activity.

The investigator or an authorized associate must explain the nature of the study and the treatment in such a manner that the subject is aware of his/her rights and responsibilities, as well as potential benefits and risks. The investigator is also responsible for answering any questions the subject may have throughout the study and for sharing any new information, in a timely manner, that may be relevant to the subject's willingness to continue his/her participation in the study. The Informed Consent Form (ICF) and Patient Information Leaflet (PIL) also include indications about the insurance coverage and the resulting regulations for the delimitation of damages. The subject will be informed that he/she should notify the investigator of any other medical measures during the study period and that he/she cannot simultaneously take part in another study.

Subjects must also be informed that participation is voluntary and that they may withdraw from the study at any time, without prejudice to their current or future care. Documentation of the discussion and the date of informed consent must be recorded in the subject's medical record.

Concerning the study data, by signing the ICF, the patient will accept that the study data may be examined by the Sponsor, the CAs, ECs, a mandated auditor and/or the study monitor in compliance with the statement of confidentiality.

Subjects or legal representative must sign and date the ICF after the nature of the study has been fully explained. A copy of the completed ICF as well as the PIL must be provided to the subject. Before its use, the PIL and ICF must meet local regulations and be approved by the EC

13.2 Ethical review/Ccompetent authority approval

Requirements for ethical review and competent authorities as set forth in Directive 2001/20/EC of the European Parliament and of the Council of 4 April 2001on the approximation of the laws, regulations and administrative provisions of the Member States relating to the implementation of good clinical practice (GCP) in the conduct of clinical trials on medicinal products for human use or other relevant local regulations for institutional review will be followed. The Protocol, ICF/PIL, Investigator's Brochure and other required documents must be approved by the EC and CA before enrolment of subjects in the study. The Sponsor must confirm that the EC is in compliance with the general standards for the composition, operation, and responsibility of an EC as set forth in ICH Guidelines for GCP. The letter of approval from the EC, the CA, as well as a list of documents reviewed, will be filed in the Investigator Site File (ISF) and a copy will be filed in the trial master file (TMF) held by the Sponsor.

Any member of the EC who is directly affiliated with this study as an investigator or as site personnel must abstain from the EC vote on the approval of the protocol. The Sponsor, in collaboration with the investigator, will be responsible for reporting to the EC and to the CA all changes in research activity, including protocol amendments, updates of Investigator's Brochures, annual safety reports, all unanticipated problems involving risks to human subjects, and study termination. The investigator will also be responsible for submitting progress reports to the EC at regular intervals appropriate to the degree of subject risk involved, but no less than once per year. Copies of all EC and CA notifications and approvals will be forwarded to the Sponsor.

The site will also apply to their Local Authority and Ethics Committee (EC) as appropriate for approval to participate in the study.

13.3 Regulatory Considerations

13.3.1 Responsibilities of the sponsor and investigators

Prior to initiating the clinical trial, the sponsor establishes and allocates all trial-related duties and functions. The sponsor ensures that all investigators are provided with instructions and a uniform set of standards for the assessment of clinical and laboratory findings, and on completing the eCRFs.

The different partners have to declare no conflicts of interest.

13.3.2 Responsibilities of the Investigators

The investigator agree with the requirements of the signed protocol. The investigator's responsibilities shall include but not be limited to:

- Knowledge of the properties of the IMP and familiarity with the appropriate use of the investigational medicinal product as described in the in the Investigator's Brochure
- Detailed knowledge of the clinical trial protocol
- To have sufficient time, an adequate number of qualified staff to conduct the trial properly and safely
- To ensure that adequate medical care is provided to a subject for adverse event related to the trial
- To ensure accuracy, completeness, and timeliness of the collected data, documents and reports
- Data reported on the eCRF should be consistent with source data
- Upon request of the monitor, auditor or regulatory authority, direct access to all trialrelated records should be permitted
- To ensure that patient's anonymity is maintained

- To declare financial interests in the clinical trial and IMP if applicable

The investigator is responsible for the conduct of the clinical trial at the respective site. In signing this protocol, the Investigator accepts to carry out all procedures related to this study according to the laws and guidelines of the EU regarding the conduct of clinical research and any local requirements of the individual EU country. Investigator must allow access to all documents pertinent to the study. In particular, the Investigator must comply with current international conference on harmonization (ICH) tripartite guidelines for good clinical practice (GCP) and current EU Directive on clinical trials (Directive 2001/20/EC of the European parliament and of the council of 4 April 2001). The study may be subject to inspection by Regulatory Authorities or Sponsor's audit and will be monitored by accredited personnel. The Protocol must be read thoroughly and the instructions herein must be followed exactly. Any deviations should be agreed between the Sponsor and the Investigator before the occurrence of the deviation, with appropriate written protocol deviations made to reflect the changes agreed upon. Where the deviation occurs for the well-being of the patient, the monitor must be informed and a course of action agreed. If the Investigator moves, withdraws from the study or retires, the responsibility for conducting the study and maintaining the records may be transferred to another Investigator at the same centre who will accept responsibility for taking over the study. Notice of transfer must be made to, and agreed by the Sponsor.

13.3.3 Patient confidentiality

Recording, transmission and storage of subjects' trial-relevant data will be performed according to local secrecy obligations, as well as national and European requirements (EU Directive 96/46 on data protection).

The principal investigator must ensure that the patient's anonymity is maintained. On the eCRFs or other documents submitted to the Sponsor, subjects should not be identified by their names, but by their assigned identification number. If patient names are included on copies of documents submitted to the Sponsor, the names must be obliterated and replaced with the assigned study patient numbers.

Participants are separately informed about data security in the patient information leaflets /informed consent form.

The principal investigator should keep a separate log of patient identification numbers, names, addresses, telephone numbers and hospital numbers (if applicable). Documents not for submission to the Sponsor, such as signed informed consent forms, should be maintained in strict confidence by the principal investigator in the ISF.

A screening failure log will be maintained for subjects who have consented to participate in the study but who, for whatever reason, are not eligible, withdrawn or decide to withdraw prior to taking part. This log will contain the following information:

- Patient study number.
- Reason for study withdrawal (when available).

eCRF pages will not be completed for these subjects.

The investigator shall permit authorised representatives of the Sponsor, regulatory authorities and IECs to review that portion of the patient's medical record that is directly related to the study. As part of the required content of informed consent, the patient must be informed that his/her records will be reviewed in this manner.

13.3.4 Good Clinical Practice

This study will be conducted in accordance with the protocol and ethical principles stated in the Declaration of Helsinki or the applicable guidelines on GCP, and all applicable local laws, rules, and regulations.

All data recorded in the case report form (eCRF) for subjects participating in this study will be transcribed from source documents.

In parallel to the submission to the EC, the Sponsor has to obtain an authorisation from the appropriate competent authority (CA) to conduct the clinical study. Subjects must not be entered into the study until the relevant EC has issued its opinion and the CA has given authorisation to conduct the study.

All substantial amendments must be submitted to the EC and/or to the CA for approval.

13.3.5 Amendments to the clinical trial protocol

The investigator should not implement any deviation from, or changes of the protocol without agreement by the sponsor or his delegate and prior review and documented approval of an amendment by the competent authority and the concerned ethics committee, except where necessary to eliminate an immediate hazard to trial participants, or when the change involves only administrative aspects, per European law (Directive 2001/20).

13.3.6 Declaration of End of trial

The end of the trial will be notified to concerned ethics committee and competent authority within 90 days, as required by European and local legislations.

13.3.7 Final Report Signature

A clinical study report, written in accordance with ICH Guideline E3, will be submitted in accordance with local regulations and requirements set forth in Directive 2001/20/EC of the European Parliament and of the Council of 4 April 2001 on the approximation of the laws, regulations and administrative provisions of the

Member States relating to the implementation of GCP in the conduct of clinical trials on medicinal products for human use.

13.3.8 Financing and Insurance

The clinical trial is financed by Ministry of Health the, under grant agreement number GR-2018-1236716**.** No funds from third parties are involved.

The Sponsor or his delegate will procure insurance by for this clinical trial to cover trial related injuries of the participants according to local regulatory requirements.

Clinical trial participants will be provided on request with the conditions of insurance provided by along with the patient information and consent form.

13.3.9 Investigator Information

The contact information and qualifications of the principal investigator and sub-investigators and name and address of the research facilities are included in the ISF.

14 Clinical trial registry

The clinical trial will be registered in an appropriate public clinical trial registry before the first participant is enrolled. The registry should meet the ICMJE criteria (e.g. DRKS, ClinicalTrials.gov).

15 Publication policy

Upon study completion and finalization of the study report the results of this trial will be submitted for publication, irrespective of findings.

16 Sponsor, coordinating centre(s) and committees

Campus Bio-Medico will be the Sponsor of the trial, the investigation being conducted in 1 countrie, Italy. The management of the study will be conducted at the local level by Sponsor/CRO with highest quality and according to national and international standards. The Sponsor is responsible for the overall study and it will guarantee that the trial is conducted correctly in compliance with the protocol, ICH-GCP (DIRECTIVE 2001/20/EC), the current version of the Declaration of Helsinki and all applicable regulatory requirements. A quality control system will be established under the responsibility of the sponsor.CRO will support the Sponsor by providing several coordinated services: regulatory and ethical submissions, monitoring, IMP management support and local pharmacovigilance provided

through the Sponsor, and data management, as a central service, provided through one of the compliant with EMA, AIFA and FDA requirements.

17. References:

- Ankrum JA, Ong JF, Karp JM. (2014) Mesenchymal stem cells: immune evasive, not immune privileged Naturebiotech, 32 (3) 252-254.

- Blanco JF, Graciani IF, Sanchez-Guijo FM, Muntion S, Hernandez-Campo P, Santamaria C, Carrancio S, Barbado MV, Cruz G, Gutierrez-Cosio S, Herrero C, San Miguel JF, Brinon JG & Del Canizo MC. (2010). Isolation and Characterization of Mesenchymal Stromal Cells From Human Degenerated Nucleus Pulposus: Comparison With Bone Marrow Mesenchymal Stromal Cells From the Same Subjects. Spine 35, 2259-2265.

- "Brinjikji W, Lehman VT, Huston J 3rd, Murad MH, Lanzino G, Cloft HJ, Kallmes DF. The association between carotid intraplaque hemorrhage and outcomes of carotid stenting: a systematic review and meta-analysis. J Neurointerv Surg. 2017 Sep;9(9):837-842. doi: 10.1136/neurintsurg-2016-012593. Epub 2016 Aug 18. Review.

Cartabellotta A, Salvioli S. Linee guida per la valutazio- ne e il trattamento di lombalgia e sciatalgia. Evidence 2017;9(4): e1000165.

- DIRECTIVE 2001/20/EC OF THE EUROPEAN PARLIAMENT AND OF THE COUNCIL of 4 April 2001on the approximation of the laws, regulations and administrative provisions of the Member States relating to the implementation of good clinical practice in the conduct of clinical trials on medicinal products for human use.

- Fang D, Seo BM, Liu Y, Sonoyama W, Yamaza T, Zhang C, Wang S, Shi S. Transplantation of mesenchymal stem cells is an optimal approach for plastic surgery. Stem Cells. 2007 Apr;25(4):1021-8. Epub 2006 Dec 14.

- Fang L, Lange C, Engel M, Zander AR, Fehse B. Sensitive balance of suppressing and activating effects of mesenchymal stem cells on T-cell proliferation. Transplantation. 2006 Nov 27;82(10):1370-3.

- FDA Drug Safety Communication: FDA strengthens warning that non-aspirin nonsteroidal anti-inflammatory drugs (NSAIDs) can cause heart attacks or strokes (2015) (https://www.fda.gov/drugs/drug-safety-and-availability/)

- Griffith JF et al (2007) Spine; Modified Pfirrmann grading system for lumbar intervertebral disc de generation.

- Henriksson HB, Svanvik T, Jonsson M, Hagman M, Horn M, Lindahl A & Brisby H. (2009). Transplantation of human mesenchymal stems cells into intervertebral discs in a xenogeneic porcine model. Spine 34, 141-148.

- Hiyama A, Mochida J, Iwashina T, Omi H, Watanabe T, Serigano K, Tamura F & Sakai D. (2008). Transplantation of mesenchymal stem cells in a canine disc degeneration model. J OrthopRes 26, 589-600.

- Hoy D et al (2014) Annals of the rheumatic diseases; The global burden of low back pain: estimates from the Global Burden of Disease 2010 study.

- Imran Ullah*, Raghavendra Baregundi Subbarao* and Gyu Jin Rho. Human mesenchymal stem cells - current trends and future prospective. Bioscience Reports (2015) 35, 1-18.

- Maher C et al (2017) Lancet; Non-specific low back pain.

- Noriega DC et al (2017) Transplantation; Intervertebral Disc Repair by Allogeneic Mesenchymal Bone Marrow Cells: A Randomized Controlled Trial

- Orozco L et al (2011). Transplantation; Intervertebral Disc Repair by Autologous Mesenchymal Bone Marrow Cells: A Pilot Study

- Orozco L, Munar A, Soler R, Alberca M, Soler F, Huguet M, Sentís J, Sánchez A, García-Sancho J. (2013) Treatment of knee osteoarthritis with autologous mesenchymal stem cells: a pilot study. Transplantation 95:1535-1541

- Orozco L, Munar A, Soler R, Alberca M, Soler F, Huguet M, Sentís J, Sánchez A, García-Sancho J. (2014) Treatment of knee osteoarthritis with

autologous mesenchymal stem cells: two-year follow up results. Transplantation 97: e66-68

- Peng BG, Pathophysiology, diagnosis, and treatment of discogenic low back pain. (2013) World J Orthop April 18; 4(2): 42-52

- Pers, YM., Rackwitz, L., Ferreira, R., Pullig, O., Delfour, C., Barry, F., Sensebe, L., Casteilla, L., Fleury, S., Bourin, P., Noël, D., Canovas, F., Cyteval, C., Lisignoli, G., Schrauth, J., Haddad, D., Domergue, S., Noeth, U., Jorgensen, C. and on behalf of the ADIPOA Consortium (2016), Adipose Mesenchymal Stromal Cell-Based Therapy for Severe Osteoarthritis of the Knee: A Phase I Dose-Escalation Trial. STEM CELLS Translational Medicine, 5: 847–856. doi:10.5966/sctm.2015-0245

- Pfirrmann CW1, Metzdorf A, Zanetti M, Hodler J, Boos N. Magnetic resonance classification of lumbar intervertebral disc degeneration. Spine (Phila Pa 1976). 2001 Sep 1;26(17):1873-8

- PopulationReferenceBureau; 2016 World population data sheet

- Serigano K, Sakai D, Hiyama A, Tamura F, Tanaka M, Mochida J. Effect of cell number on mesenchymal stem cell transplantation in a canine disc de generation model. J Orthop Res. 2010 Oct;28(10):1267-75. doi: 10.1002/jor.21147

- Vadala G et al (2016) World journal of stem cells; Stem cells sources for intervertebral disc re generation

- Vadala G, Russo F, Di Martino A, et al. Intervertebral disc regeneration: from the degenerative cascade to molecular therapy and tissue engineering. J Tissue Eng Regen Med. 2015;9(6):679-90.

- Vadala G, Studer RK, Sowa G, Spiezia F, Iucu C, Denaro V, Gilbertson LG & Kang JD. (2008). Coculture of bone marrow mesenchymal stem cells and nucleus pulposus cells modulate gene expression profile without cell fusion. Spine 33, 870-876

- Vega A, Martin-Ferrero MA, Del Canto F, Alberca M, Garcia V, Munar A, Orozco L, Soler R, Fuertes JJ, Huguet M, Sanchez A & Garcia-Sancho J.

(2015). Treatment of Knee Osteoarthritis With Allogeneic Bone Marrow Mesenchymal Stem Cells: A Randomized Controlled Trial. Transplantation 99, 1681-1690

- WHO (2013) Priority medicines for Europe and the World "A public health approach to innovation". Background Paper 6.24 - Low back pain

- Yang SH, Wu CC, Shih TT, Sun YH & Lin FH. (2008). In vitro study on interaction between human nucleus pulposus cells and mesenchymal stem cells through paracrine stimulation. Spine 33: 1951-1957
